# Human hepatoma Huh-7 cell culture models deficient in apolipoprotein B secretion

**DOI:** 10.1016/j.jlr.2025.100867

**Published:** 2025-07-23

**Authors:** Swati Valmiki, Sara Rosario, Ashley Mooring, Chandana Prakashmurthy, Florencia Schlamp, Binu Prakash, Atrayee Chattopadhyay, Abulaish Ansari, José O. Alemán, Nathalie Pamir, M. Mahmood Hussain

**Affiliations:** 1 Department of Foundations of Medicine, NYU Grossman Long Island School of Medicine, Mineola, NY; 2 Oregon Health and Science University, Portland, OR; 3 Laboratory of Translational Obesity Research, Holman Division of Endocrinology, NYU Langone Medical Center, New York, NY; 4 Leon H. Charney Division of Cardiology, Department of Medicine, Cardiovascular Research Center, New York University School of Medicine, New York City, NY

**Keywords:** apoB100, lipid transport, CRISPR-Cas9, DNA replication, coagulation, vitamin D binding protein

## Abstract

ApoB is an essential structural protein for the assembly and secretion of triglyceride-rich lipoproteins and therefore remains a potential target to lower plasma cholesterol levels in hypercholesterolemia patients. To understand the global consequences of *APOB* gene deficiency, we employed CRISPR-Cas9 system to generate apoB-deficient human hepatoma Huh-7 cells (Ako cells). ApoB was not detectable in the cells and media of the Ako cells. ApoB deficiency had no effect on microsomal triglyceride transfer protein expression and activity. These cells supported apoB48 secretion when transfected with plasmids for the expression of apoB48 suggesting that these cells retain all the lipoprotein assembly and secretion machinery except for apoB expression. *APOB* gene deficiency had no significant effect on cellular lipid levels, cell growth, and ER stress markers. Proteome analysis of secreted proteins revealed that the most upregulated protein was the vitamin D binding protein, while the most downregulated protein was apoB in Ako cells compared to control cells. This analysis also identified coagulation as an upregulated pathway. Total RNA transcriptome analysis identified DNA replication and complement and coagulation pathways as the most upregulated pathways in Ako cells. Further detailed studies are needed to establish how apoB regulates these pathways. These Ako cells may be useful in studying structure-function analysis of apoB peptides and to address the cellular consequences of disruptions in lipoprotein assembly and secretion.

Transport of dietary and endogenous fat in vertebrates depends on the assembly and secretion of triglyceride (TG)-rich apoB-containing lipoproteins, chylomicrons, and VLDLs by the intestine and liver, respectively. Maintenance of normal function and plasma concentrations of these lipoproteins are critical for normal physiology as high plasma concentrations of these lipoproteins are risk factors for various metabolic diseases, such as atherosclerotic cardiovascular disease, obesity, and diabetes (1, 2). Increasing the clearance of these lipoproteins from plasma has been and continues to be a successful and preferred strategy to reduce plasma lipids and atherosclerosis. Despite these successes, premature heart disease mortality is almost 1-in-5 of all deaths (3). Therefore, additional approaches are needed to reduce morbidities and mortalities associated with heart diseases. A complementary approach to reducing plasma lipoproteins is to curtail their hepatic and intestinal production. Since apoB lacks a functional assay, a predominant approach has been to reduce apoB mRNA using antisense approach (4, 5, 6). Another approach to reduce plasma lipoprotein production has been to inhibit the function of an obligatory protein, microsomal triglyceride transfer protein (MTP), for the assembly and secretion of apoB-containing lipoproteins (7, 8). Inhibition of these proteins reduces plasma lipid and lipoprotein concentrations; however, these strategies are associated with lipid accumulation in tissues (4, 5, 8). Hence, there is a need to understand global consequences of significant reductions in the synthesis of apoB-containing lipoproteins and understand molecular and physiological consequences of inhibiting apoB-containing lipoprotein biosynthesis. An approach to obtain such information is to characterize patients, animals, and cell culture models deficient in the production of apoB-containing lipoproteins.

Human *APOB* gene deficiency in familial hypobetalipoproteinemia-secretion defect 2 or homozygous hypobetalipoproteinemia results in significant reductions in plasma lipids and fat-soluble vitamins (9, 10). Although familial hypobetalipoproteinemia-secretion defect 2 patients have been studied for disease progression and health maintenance, global changes in their tissue transcriptome have not been studied and are difficult to obtain for ethical reasons. Attempts to understand the roles of *APOB* gene in mice have been hampered as knockout of this gene leads to embryonic lethality (11). Hence, there is a need to develop cell culture model systems to study the role of apoB in hepatic lipid metabolism.

Recent advances in CRISPR/Cas9 approaches have allowed generation of different gene deficient cell culture models to study physiological roles of candidate proteins. Previously, we reported the generation of MTP-deficient human hepatoma cells and showed that these cells were defective in lipid and apoB secretion (12). Similarly, apoB and MTP-deficient human intestinal carcinoma Caco-2 cells have been shown to be defective in lipid and lipid-soluble vitamin absorption (13). Here, we report successful establishment of *APOB* gene ablated Huh-7 (Ako) cells that are deficient in secreting apoB-containing lipoproteins. These cells, however, secrete apoB48 when transfected with expression plasmids. Different pathway analyses following total RNA transcriptome sequencing identified DNA replication and complement and coagulation as the most upregulated pathways.

## Materials and Methods

### Materials

Single guide RNA (sgRNA) and SpCas9 nuclease were designed and obtained from Synthego. Primers used for PCR amplification and sequencing were ordered from Integrated DNA Technology. NBD-TG (1,3-Diolein, 2-NBD-X ester, #6285) was from Setareh Biotech. NBD-phosphatidylethanolamine (#810145P), L-α phosphatidylcholine (Egg PC, #131601C), and 1,2-dioleoyl-sn-glycero-3-phosphoethanolamine (# 850725C) were from Avanti Polar Lipids. Rabbit polyclonal anti-MTP antibody was from Abcam (#ab63467) and rabbit polyclonal anti β-actin primary antibody was from Cell Signaling (#4967S). Vitamin D binding protein (VDBP) polyclonal primary antibody was from Invitrogen (#PA5-29082). cDNA synthesis kit (Applied Biosystems, #A46109), and quantitative PCR (qPCR) kits were from Eurogentec (# RT-SN10-05). Round-bottom black 96-well assay plates were obtained from Costar Kennebunk, ME (#3792).

### Growth and maintenance of Huh-7 cells

Huh-7 cells were grown and maintained in DMEM supplemented with 10% FBS and L-Glutamine at 37°C and 5% CO_2_ in humidified incubator as described previously (12, 14, 15, 16, 17). Cells were grown until they reached 80–90% confluence with media changed at regular intervals. For fatty acid supplementation studies, cells were grown and maintained in DMEM with 0.2 mM oleic acid (OA) complexed with 1.5% BSA throughout the study.

### Guide RNA design, ribonucleoprotein complex formation, and transfection into Huh-7 cells

To carry out *APOB* gene deletion in Huh-7 cells, synthetic guideRNAs (gRNAs) were designed using Synthego’s CRISPR design tool. We used Synthego’s guide verification tool to minimize the possibility of off-target effects of selected sgRNAs. Furthermore, the sgRNAs were designed based on the availability of PAM sequences in specific genomic locations, making them more specific for target gene. Top three gRNAs with minimum off-target effects gRNA1-AGCCCACUUGCUCUCAUCAA, gRNA2-AUAUCCACUGAAAGAGACCU, and gRNA3-CCUCCAUAGGAUACCGUGUA were purchased from Synthego. These gRNAs targeted 160 bp region within the exon 6 of the *APOB* gene. The gRNA and Cas9 ribonucleoprotein (RNP) complexes were prepared and transfected in Huh-7 cells by electroporation as described earlier (12). Briefly, a mixture of three different gRNA (10 μM each) and SpCas9 Nuclease (20 μM) were mixed in 7 μl PBS and incubated for 15 min at room temperature. Huh-7 cells grown to 80–90% confluence in T75 flasks were trypsinized and washed three times with PBS with centrifugation at 1,250 *g* for 5 min at room temperature. Finally, 10^6^ cells were resuspended in 5 μl of buffer R (Neon Resuspension Buffer R #MPK10096R). The RNP complex and Huh-7 cells were mixed and 10 μl of this mixture was aspirated into gold plated tips using pipette supplied with the kit (Thermo Fisher Scientific # MPK1096) and exposed to 1650V for 10 ms using Neon transfection system (Thermo Fisher Scientific, #MPK5000) as per the manufacturer protocol. Electroporated cells were immediately transferred to 6 well plates containing DMEM and allowed to grow at 37°C in CO_2_ incubator until they reached 80–90% confluence with media changed every 48 h. The Huh-7 cells transfected with RNP complex were trypsinized after they reached 90% confluence. Cells were divided into three parts; one part was used for genomic DNA isolation and PCR amplification to confirm gene ablation. The second part was serially diluted and transferred to 96-well plates to obtain single cell per well, and the third part was frozen and stored at −80°C for further use.

### Confirmation of gene ablation and colony expansion

To confirm gene ablation, the gRNA target sites within exon 6 of the *APOB* gene were PCR amplified using upstream forward primer 1 (5′ TCTGCTTTTTCTTTCACGATCCC 3′) and downstream reverse primer 1 (5′ GCAGGCTGTCCTCAAAGGTG 3′) (Supplementary Fig. 1A). The PCR products were resolved and visualized by performing agarose gel electrophoresis, purified from gels using DNA clean up kit (BioLabs, #T1030S), and sequenced using the forward primer 1 and reverse primer 1 (Genewiz). The chromatograms received after sequencing were aligned with the reference sequence and analyzed using SnapGene software.

### RNA isolation and quantitative reverse transcription polymerase chain reaction (qRT-PCR)

RNA was isolated from cells grown in 6-well plates using Trizol reagent (Thermo Fisher Scientific, #15596018) following manufacturers protocol. Nanodrop spectrophotometer (Thermo Fisher Scientific, #ND-ONE-W) was used for the quantitative and qualitative assessment of RNA. cDNA synthesis was carried out using 2 μg RNA and random primer with SuperScript IV Cells Direct cDNA Synthesis Kit (Invitrogen, #11750150). Gene expressions were measured by performing qRT-PCR using the 2X qPCR kit (Eurogentec, #RT-SN10-05) and Real-time PCR machine QuantStudio3 (Applied Biosystems, #A28131). Ten times diluted cDNA was used as template for the qPCR reaction and 18S was used as internal reference. The primers used for amplifications are in Supplemental Table 4.

### Intracellular and media apoB detection by ELISA

ApoB protein expression was quantified by ELISA (18, 19). Cells grown in 6-well plates to 80–90% confluence were used to detect intracellular apoB expressions and media to quantify secreted apoB. ELISA 96-well plates were coated with 100 μl of mouse monoclonal anti-hApoB 1D1 (capture antibody, 1:1000 dilution in PBS, My BioSource, # MBS465020) by overnight incubation at 4°C. Next morning, plates were washed three times with 200 μl PBS with 0.05% Tween 20 (PBST) and blocked in 3% BSA prepared in PBS for 1 h at 37°C. Next, plates were washed three times with PBST and 100 μl of diluted or undiluted conditioned media was added to different wells along with LDL standards with a concentration of 0–100 ng/ml and incubated at 37°C for 2 h. Plates were washed three times with PBST and incubated with 100 μl of polyclonal anti-hApoB detection antibody (1:1000 dilution in PBST, Academy Bio-medical Company, #20S-G2) for 1 h at 37°C. After washing three times with PBST, 100 μl of alkaline phosphatase–labeled swine polyclonal anti-goat IgG antibody was added (1:1000 dilution in PBST, Southern Biotech, #6300-04) for 1 h at 37°C. Finally, plates were washed three times with PBST and 100 μl of 1 mg/ml (w/v) of p-nitrophenyl phosphate substrate prepared in diethylamine buffer (100 mM glycine, 1 mM MgCl_2_.6H_2_O, and 1 mM ZnCl_2_, pH 10.4) was added to develop yellow color. Absorbance was measured at 405 nm and apoB100 concentration in the samples was calculated using LDL standard curve generated in parallel in the same plate and subjected to linear regression analysis (18). To detect intracellular apoB100 protein expression, cells were washed with PBS, harvested in RIPA buffer (Thermo Fisher Scientific, #J63306), and centrifuged at 12,000 rpm or 13,500 *g* for 10 min at 4°C to remove cellular debris. The clear supernatant obtained after centrifugation was used for apoB100 detection by ELISA. An aliquot of cellular homogenate was also used for protein estimation using Pierce BCA protein estimation kit (Thermo Fisher Scientific, #23225) and this value was used to normalize the apoB100 concentrations in cells and conditioned media.

### Western blot for protein expression

To check for protein expression, Huh-7 and Ako cells grown in 6-well plates were harvested in 1× RIPA buffer (Thermo Fisher Scientific #J63306) and centrifuged at 12,000 rpm or 13,500 *g* for 10 min at 4°C to remove cellular debris. From the top clear supernatant, 25 μg protein was used to detect MTP and apoB48 and 40 μg protein was used to detect apoB100. Secreted apoB and VDBP were quantified in the overnight conditioned media (40 μl) by Western blotting after separating on gels. The cellular homogenates and media from respective wells were mixed with 4× sample buffer (Bio-Rad, #1610747) and resolved on SDS-PAGE (6% for apoB, 8% for MTP and 10% for VDBP) followed by transfer to nitrocellulose membrane. Membranes were probed with rabbit polyclonal anti-hMTP primary antibody (Abcam, #ab63467) to detect MTP, mouse monoclonal anti-hapoB 1D1 antibody (My BioSource, #MBS465020) to detect apoB, VDBP polyclonal primary antibody (Invitrogen # PA5-29082) to detect VDBP, and rabbit polyclonal anti β-actin primary antibody (Cell Signaling, #4967S) to detect β-actin, which was used as loading control. Anti-rabbit IgG HRP (Cell Signaling, #7074S) was used as secondary antibody for MTP, VDBP, and β-actin. Goat anti-mouse IgG HRP (Invitrogen #62-6520) was used as secondary antibody for apoB.

### MTP lipid transfer activity

The donor and acceptor vesicles to study TGs and phospholipid (PL) transfer were prepared as described earlier (20, 21, 22). To study lipid transfer activity of MTP, Huh-7 and Ako cells grown in 6-well plates were harvested in buffer K (1 mM Tris–HCl, 1 mM EGTA, 1 mM MgCl_2_, pH 7.6) and lysed by sonication (40% amplitude, 2 s pulses on and 1 s off, for 90 s) using Branson Digital Sonifier SFX 150. Cells were centrifuged at 12,000 rpm or 13,500 *g* for 10 min at 4°C to remove dead cells and cellular debris. From the clear supernatant, 30 μg protein was used to measure TG and 50 μg protein was used for PL transfer activities (20, 21, 23, 24). For TG transfer assay, cellular homogenates were incubated with a mixture of donor vesicles containing NBD-TG and acceptor vesicles. For PL transfer assay, cellular homogenates were incubated with donor vesicles containing NBD-phosphatidylethanolamine and acceptor vesicles containing phosphatidylcholine. For blank fluorescence measurements, vesicles were in buffer K. Total fluorescence was obtained by disrupting the vesicles with isopropanol. Percent TG and PL transfer were calculated by subtracting the blank, dividing it by total fluorescence, and multiplying with 100 (20, 21, 23).

### Transgenic apoB48 expression and fractionation of secreted lipoproteins

The ability of Ako cells to support assembly and secretion of transgenic apoB was studied by transfecting cells with plasmid expressing human apoB48 (15, 16, 25) or empty vector pcDNA as negative control. Ako cells were seeded in 6-well plates at a density of 150,000 cells per well. After 24 h, cells were transfected with 3 μg of plasmid expressing either apoB48 or empty vector pcDNA (control). After 36 h, media was replaced with fresh media for overnight incubation and apoB48 quantification. Next morning, conditioned media was collected from the 6-well plates and apoB48 was detected in media by performing apoB ELISA and Western blot analysis. Overnight conditioned media (40 μl) was resolved on 6% polyacrylamide gel and probed with mouse monoclonal anti-hApoB monoclonal 1D1 antibody to detect apoB48 protein.

To characterize the secreted lipoproteins, overnight conditioned media from parent Huh-7 cells expressing apoB100 and Ako cells expressing transgenic apoB48 were used to separate the plasma lipoproteins by AKTA pure fast-protein liquid chromatography (FPLC). Ako and Huh-7 cells were seeded in 150 cm^2^ dishes at density of 900,000 cells per dish. After 24 h, Ako cells were transfected with 20 μg pf plasmid for the expression of human apoB48 and allowed to grow until reached 80–90% confluence. After 36 h, the media was replaced with fresh media for overnight incubation in both Huh-7 and Ako cells. Next morning, the overnight conditioned media was collected and concentrated using 10 kD centrifugal filter units (Merck #UFC901024) until the volume was reduced from 12 ml to 500 μl. The concentrated media (250 μl) was applied to a FPLC column [Superose™ 6 Increase 10/300 Gl FPLC column (GE Healthcare)], eluted with PBS at a flow rate of 0.4 ml/min, and 0.25 ml fractions were collected. ApoB was measured in the collected fractions by ELISA.

### TG accumulation in cells

Huh-7 and Ako cells were seeded at a density of 10^5^ cells per well in 6-well plates and allowed to grow for 96 h with media changed every 24 h. Cells were then used to quantify TG after lipid extraction or Oil Red O staining. For total lipid extraction, cells were washed with PBS three times, 1 ml of 100% isopropanol was added to the plates and incubated overnight at 4°C. Next morning, the isopropanol was collected in centrifuge tubes, dried under vacuum, and then total lipids were resuspended in 100 μl of isopropanol. To quantify TGs, 10 μl sample and 90 μl of TGs reagent (Pointe Scientific) was added in 96-well plates, incubated at 37°C for 5 min, and absorbance was measured at 490 nm.

For Oil Red O staining, Ako and Huh-7 cells were seeded and maintained in 6-well plates at a density of 10^5^ cells per well with media changed at 24 h. After 96 h, media was aspirated form the wells, cells were washed three times with PBS, and 2 ml of 10% formalin was added to cell for 5 min. The 10% formalin was removed and fresh 10% formalin was added to cells. Cells were kept in formalin for 1 h and after that cells were washed three times with PBS. For staining, 2 ml of Oil Red O stain (0.36% in 60% isopropanol) was added to cells for 30 min. Cells were washed three times with PBS to remove excess stain and images were captured using Nikon Eclipse TE300 microscope under 20× magnification. After taking images, total lipids were extracted from the wells by adding 1 ml isopropanol and absorbance was measured at 490 nm.

### RNA-Seq and differential expression analysis

To understand the consequences of *APOB* gene ablation on transcriptome, mRNA Libraries were prepared and sequenced by Novogene, on Illumina NovaSeq X plus Series (PE150) as paired end reads. The raw reads were processed and aligned to the reference genome hg38 using HISAT2 (v2.0.5) and have been deposited (GSE269949 for Ako cells and Mko cells). FeatureCounts (v1.5.0-p3) was used to produce gene-level count tables. Differential analysis was performed using DESeq2 (1.20.0).

### Proteome analysis of secreted proteins

Proteomic and lipidomic analyses of the secretome were performed to identify the changes in total secretory proteins and lipids. Huh-7 and Ako cells were grown in T75 flasks until reached 80–90% confluence. Cells were washed three times with PBS to remove FBS and incubated overnight (16 h) with serum-free DMEM. The overnight conditioned media was collected, protease inhibitor was added, and the media was concentrated using 10 kD centrifugal filter units (Merck #UFC901024). The concentrated media was used for proteomic (26, 27) analyses.

For proteomic, proteins were measured using bicinchoninic acid and proteins (1 μg) were solubilized in 0.1% RapiGest (Waters) in 100 mM ammonium bicarbonate, reduced with dithiothreitol, alkylated with iodoacetamide, and digested overnight with trypsin (1:10, protease:protein ratio) at 37°C as described before (26, 27, 28). Tryptic digests were reconstituted in 5% acetonitrile and 0.1% formic acid and separated over a period of 90 min using a linear gradient of 0.1% formic acid in water and 0.1% formic acid in acetonitrile using an Acclaim PepMap nanoLC C18 75 μm × 25 cm column. LC-MS/MS data was acquired and analyzed using Skyline (29). Differential expression analysis for proteome data was performed using edgeR.

### STRING analysis for apoB-related protein network

Total RNA transcriptomics and proteomics data were used to identify apoB-related protein network using STRING database. For the analysis, we used basic settings such as network type: full STRING network, meaning of network edges: evidence, minimum required confidence score was set as high confidence (0.700), and maximum number of interactions to show selected for query proteins only. Further the protein clustering was done using MCL clustering option with inflation parameter set as 3.

### Cell proliferation assay

The cell proliferation assay was performed using Cell Titre 96 Aqueous One Solution Cell Proliferation Assay (Promega #G3582) using manufacturer’s protocol. Briefly, Ako clones and Huh-7 cells were seeded at a density of 2000 cells per well in 96-well plates and maintained as described earlier. For lipid stimulated conditions, both Ako and Huh-7 cells are grown and maintained in DMEM supplemented with 10% FBS and 0.2 mM OA (Sigma #O7501). Cell growth was monitored at different time points; 24 h, 48 h, 72 h, and 96 h. For this, cells were incubated with assay reagent for 3 h at 37°C at different time points and the absorbance was measured at 490 nm.

### Statistical analysis

Data are presented as the means ± SD. Significance of differences between two groups were calculated using the GraphPad Prism software (GraphPad Software, San Diego, CA). Statistical significance (∗*P* < 0.05, ∗∗*P* < 0.01, and ∗∗∗*P* < 0.001) was determined using Student's *t* test (GraphPad Prism 9), multiple *t* tests or two-way ANOVA for grouped analyses. The significance level of the differences of the group was indicated in the legend of each figure.

## Results

### *APOB* gene ablation leads to loss of apoB secretion in human hepatoma cells

The human hepatoma Huh-7 cells express MTP and apoB100 and support the assembly and secretion of apoB100-containing lipoproteins (14, 15, 30). In the current study, we deleted the *APOB* gene in Huh-7 cells using gRNA and Cas9 nuclease protein (RNP) complexes (12). For gene deletion, three gRNAs with minimum off target effects were selected. These gRNAs targeted the 160 bp region within the exon 6 of the *APOB* gene (Supplementary Fig. 1A). The Huh-7 cells were electroporated with RNP complexes to induce insertions and deletions (indels) (Supplementary Fig. 1B, C). Primary screening for *APOB* gene deficiency was performed by quantifying apoB100 in the media. Cells transfected with RNP complexes did not support apoB100 secretion as evidenced by the absence of apoB in media compared with the control Huh-7 cells (Supplementary Fig. 2A). Second, we looked for changes in targeted DNA regions for indels. A single intact band was observed at 358 bp in the control cells, whereas the gRNA/Cas9 electroporated cells showed smaller DNA bands indicating for indels (Supplementary Fig. 2B). These studies indicated that selected gRNAs cause changes in DNA and reduce secretion of apoB and encouraged us to obtain cell clones deficient in *APOB* gene expression.

### Selection and propagation of *APOB* gene–deficient cell clones

**Figure 1:**
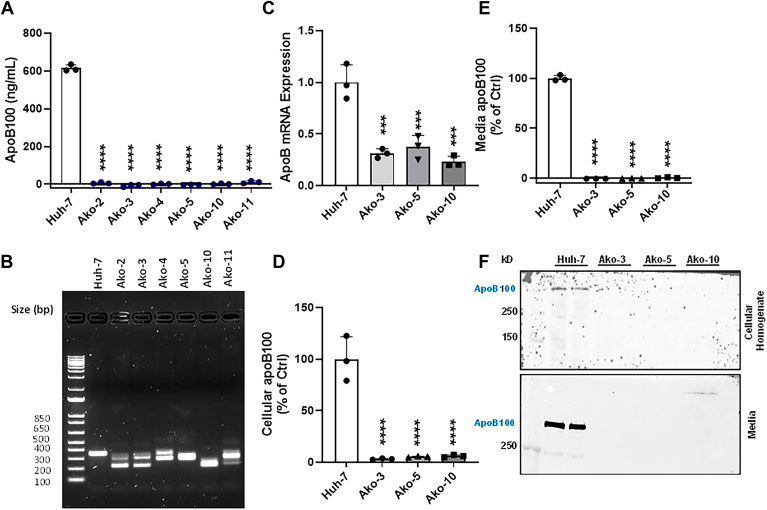
*APOB* gene ablation decreases apoB mRNA and protein levels. A: Ako and control Huh-7 cells were grown and maintained in DMEM with 10% FBS and L-glutamine in 12-well plates with media changed every 48 h. After one week, overnight conditioned media was collected from the six selected Ako clones and control Huh-7 cells to quantify apoB100 by ELISA. B: Genomic DNA was isolated from different clones, the targeted regions within the exon 6 of *APOB* gene were PCR amplified using forward and reverse primers and resolved on 2% agarose gel. C: Huh-7 and Ako cells were seeded in 6-well plates at a density of 2 × 10^5^ cells per well and after 48 h, total RNA was isolated to quantify apoB100 mRNA levels by quantitative RT-PCR. ∗∗∗ and ∗∗∗∗ represent *P* < 0.001 and *P* < 0.0001, respectively. D and E: To quantify cellular apoB100, cells were harvested in RIPA buffer and centrifuged at 13,500 *g* for 10 min at 4°C to remove cellular debris. Cellular homogenates (10 μl) were used to assess apoB100 protein expression by ELISA (D). ApoB100 concentrations were normalized with total cellular protein. ApoB100 protein expression was checked in overnight conditioned media (100 μl) by performing ELISA (E). The bars and error bars represent mean ± SD. To calculate the significance one-way ANOVA nonparametric (multiple comparison) was used, ∗∗∗ and ∗∗∗∗ represent *P* < 0.001 and *P* < 0.0001, respectively. The data are representative of three independent experiments performed with biological triplicates. F: For Western blot analysis, 40 μg protein from the cell homogenates (top) and 40 μl from media (bottom) were resolved on 6% polyacrylamide gel, transferred to membranes and probed with mouse monoclonal anti-human apoB 1D1 antibody to detect apoB100.

Cells transfected with RNP complexes were subjected to limited dilutions to distribute about one cell per well in 96-well plates for clonal selection (12). The 96-well plates were regularly monitored under microscope for colony growth and visually for changes in media color. After 4 weeks, nine *APOB* gene–deficient Ako colonies were transferred to 12-well plates. Out of the nine colonies, three did not grow. After one week, the media was collected from the remaining six colonies to quantify apoB100 by ELISA. ApoB100 was not detectable in the media of all six clones (Fig. 1A). To identify mutations in the *APOB* gene, we amplified the gRNA-targeted region in the exon 6 and resolved the PCR products on 2% agarose gel. A single intact band at 358 bp was in the control Huh-7 cells but the Ako clones had multiple or lower molecular weight bands (Fig. 1B). Sequencing showed the presence of indels in all six clones. Clones 2, 4, and 11 showed indels in regions which were far away from the predicted gRNA target site (data not shown). Clone 3 showed a few point mutations at the gRNA1 target site and deletion in between the gRNA2 and gRNA3 target sites (Supplementary Fig. 2C). Clone 5 showed indels between gRNA1 and gRNA2 target sites and a few point mutations at gRNA3 site. Clone 10 showed small sequence deletion just before the gRNA1 target site and a large deletion between gRNA1 and gRNA3 target sites (Supplementary Fig. 2C). Based on the sequencing data that showed extensive indels and deletions, we selected three clones for further characterization.

### *APOB* gene ablation decreases apoB mRNA and protein levels

It is well established that the CRISPR-Cas9–mediated gene knockout approach causes indels in DNA sequence which results in frame shift mutations and premature termination codons. Incorporation of premature termination codons results in non-sense mediated mRNA decay (31). To gather insight into the *APOB* transcript levels in our knockout cells, we quantified mRNA levels by qRT-PCR in the Ako and control Huh-7 cells. The *APOB* transcript levels were significantly reduced (∼70%) in the Ako cells as compared to the Huh-7 cells (Fig. 1C). Next, we looked at the intracellular and media apoB100 protein levels in these clones by ELISA. The Ako clones showed >95% reduction in the intracellular and secreted apoB100 protein levels (Fig. 1D, E). Furthermore, Western blot analysis showed significant depletion of intracellular and media apoB100 protein in the Ako clones (Fig. 1F). From these findings, we concluded that the CRISPR-Cas9–mediated ablation of the *APOB* gene resulted in a significantly lower levels of apoB mRNA and almost complete loss of detectable apoB protein in cells and media.

We next performed cell proliferation assay to monitor the effect of *APOB* loss on growth rate in the presence of OA to mimic the conditions of high lipid availability and in the absence of OA representing normal culture conditions. Compared to control cells, all the three Ako clones showed similar growth rates under normal and OA supplemented conditions (Supplementary Fig. 2D, E) suggesting that apoB deficiency has no significant effect on cell proliferation.

### MTP expression and lipid transfer activities are not affected by apoB deficiency

**Figure 2:**
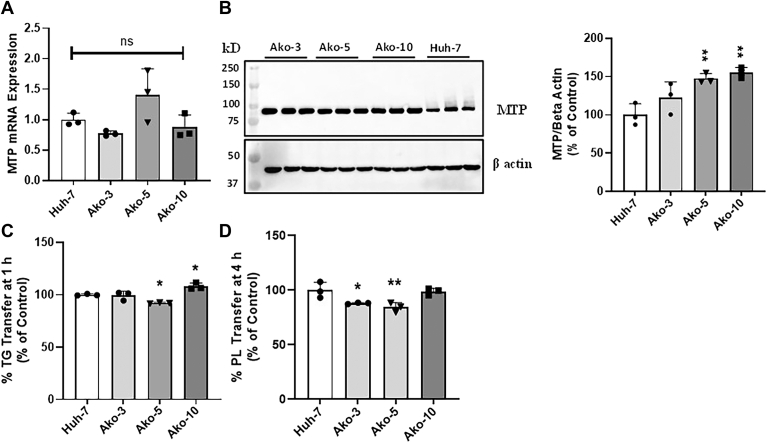
MTP expression and lipid transfer activity is not affected in the Ako clones. A: Total RNA from Huh-7 controls and Ako cells was isolated by Trizol method and used to measure relative MTP mRNA levels. B: Huh-7 control and Ako cells were harvested in RIPA buffer, centrifuged at 13,500 *g* for 10 min to remove cellular debris. For Western blotting, 20 μg protein was resolved on 8% polyacrylamide gel, transferred to membranes, and probed with polyclonal rabbit anti-MTP antibody to detect MTP or rabbit polyclonal anti β-actin antibody to detect the internal control β-actin. ∗, and ∗∗ represent *P* < 0.05 and *P* < 0.01, respectively. C and D: To quantify triglyceride (C) and phospholipid (D) transfer activities of MTP, cells were harvested in buffer K, sonicated, and centrifuged at 13,500 *g* for 10 min at 4°C to remove cellular debris. The top clear supernatant (25 μg protein) was used to perform different lipid transfer assays. MTP, microsomal triglyceride transfer protein.

MTP plays a critical role in the assembly and secretion of apoB-containing lipoproteins by transferring lipids to the nascent apoB (7, 32). Loss of MTP leads to proteasomal degradation of newly synthesized apoB resulting in the absence of media apoB. To eliminate the possibility that the absence of cellular and media apoB in our knockout cells was due to the loss of MTP, we checked the MTP transcript and protein levels in Ako cells by qRT-PCR and Western blot, respectively. Similar MTP transcript levels were observed in the Ako and Huh-7 cells (Fig. 2A). Western blot analysis showed that MTP protein levels were either unchanged or increased (Fig. 2B). Furthermore, we analyzed the lipid transfer activities of MTP by performing TG and PL transfer assays. The Ako clones efficiently transferred both lipids and these transfer activities were similar compared to those in Huh-7 cells (Fig. 2C, D). Findings from these experiments showed that the absence of apoB100 protein in the Ako cells was not due to defects in MTP expression and function and apoB deficiency does not affect MTP expression.

### Ako cells support the assembly and secretion of apoB48 after transgenic expression

**Figure 3:**
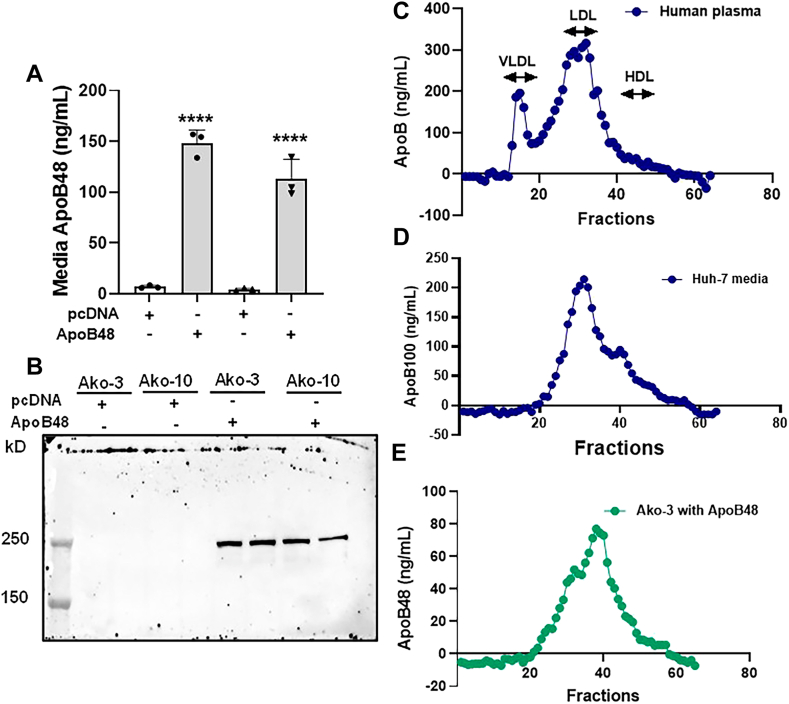
Ako cells support the assembly and secretion of apoB48 after transgenic expression. A and B: Ako cells were seeded in 6-well plates at a density of 150,000 cells per well. After 24 h, cells were transfected with 3 μg of plasmid expressing either apoB48 or empty vector pcDNA (control). After 36 h, media was replaced with fresh media for overnight incubation and apoB48 quantification. A: ApoB48 was detected in media by performing apoB ELISA. ∗∗∗∗*P* < 0.0001. B: Media (40 μl) was resolved on 6% polyacrylamide gel and probed with mouse monoclonal anti-hApoB monoclonal 1D1 antibody to detect apoB48 protein. ApoB48 was detectable in the media of cells transfected with apoB48 expression plasmids. C: Normal human plasma (250 μl) was applied to FPLC at a flow rate of 0.4 ml/minute and eluted with PBS. D and E: To characterize secreted lipoproteins by FPLC, Ako and Huh-7 cells were seeded in 150 cm^2^ dishes at a density of 900,000 cells per dish. After 24 h, Ako cells were transfected with 20 μg of plasmid expressing apoB48. After 36 h of transfection, media was replaced with fresh media for overnight incubation in both Huh-7 (D) and Ako (E) cells. The overnight conditioned media was concentrated, injected into FPLC column and eluted with PBS at a flow rate of 0.4 ml/min and 0.25 ml fractions were collected. ApoB was measured in the collected fractions by ELISA. FPLC, fast-protein liquid chromatography.

We then asked whether any other factors needed for apoB secretion were defective in Ako cells. If loss of apoB secretion is only due to the *APOB* gene deletion, we hypothesized that transgenic overexpression of the human apoB48 may result in the synthesis and secretion of apoB48. To test this, we transfected the Ako cells with pcDNA plasmids for the expression of apoB48 under the control of cytomegalovirus promoter. Plasmids expressing apoB100 are too large and difficult to express (19). Ako cells transfected with the control pcDNA plasmid had very low levels of apoB in the media (Fig. 3A). In contrast, cells transfected with plasmids for the expression of human apoB48 supported apoB48 secretion. Western blot analysis also showed a robust increase in apoB48 secretion in the Ako cells transfected with apoB48 expression plasmid compared to pcDNA transfected cells (Fig. 3B). These studies indicated that Ako cells contain all the machinery needed for the assembly and secretion of apoB.

Next, we performed FPLC analysis of overnight conditioned media to characterize secreted lipoproteins. For control, human plasma was subjected to FPLC and VLDL and LDL peaks were identified (Fig. 3C). The secreted apoB100 from control Huh-7 cells eluted at position like human LDL (Fig. 3D). Similarly, transgenic apoB48 was also secreted as LDL-size lipoprotein particle (Fig. 3E); however, the peak is shifted to the right indicating smaller apoB48 particles. Thus, Ako cells retain the capacity to assemble and secrete apoB-containing lipoproteins.

### *APOB* gene knockout does not cause fat accumulation in cells

**Figure 4:**
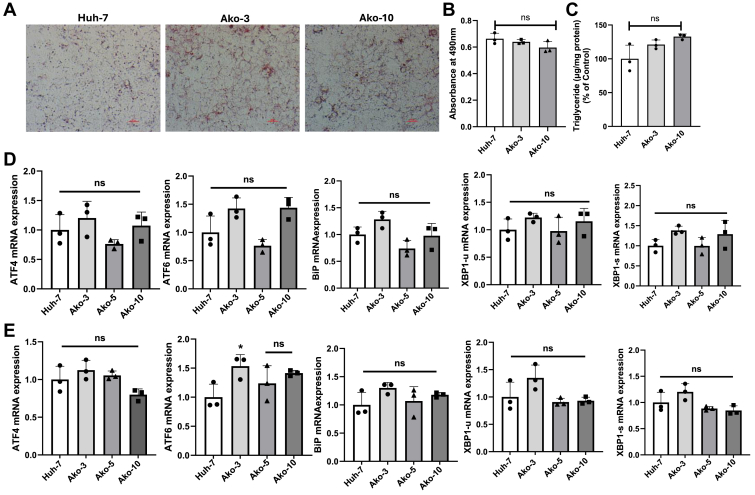
*APOB* gene knockout does not cause fat accumulation and unfolded protein response in cells. A and B: Huh-7 and Ako cells (10^5^) were seeded in 6-wells plates and allowed to grow for 96 h with media changed every 24 h. After 96 h, triglyceride accumulation in cells was quantified after isopropanol extraction and via Oil Red O staining. Cells were stained with Oil Red O for 30 min. After three washes images were captured using Nikon Eclipse TE300 microscope under 20× magnification, scale bar, 100 pixels (A). After taking images, Oil Red O stain was extracted from the wells by adding 1 ml isopropanol and absorbance was measured at 490 nm (B). C: For total lipid extraction, 1 ml of 100% isopropanol was added to the plates and incubated overnight at 4°C. Next morning the isopropanol was collected in centrifuged tubes, completely dried under vacuum, and then total lipids were resuspended in 100 μl of isopropanol. To quantify triglycerides, 10 μl of samples and 90 μl of triglyceride reagent (Pointe Scientific) were loaded in 96-well plates, incubated at 37°C for 5 min, and absorbance was measured at 490 nm. The bars and error bars represent mean ± SD. To calculate the significance, one-way ANOVA nonparametric (multiple comparison) was used. D: Ako and Huh-7 cells were seeded in 6-well plates at density of 2 × 10^5^ cells per well. After 48 h, when cells were confluent, total RNA was isolated by Trizol method and qPCR was performed to measure relative mRNA levels. E: Ako and Huh-7 cells were seeded at a density of 2 × 10^5^ cell per well in 6-well plates with DMEM containing 0.2 mM OA complexed with 1.5% BSA. After 48 h, total RNA was isolated with Trizol method and mRNA levels of genes involved in unfolded protein response was studied. Statistical significance was measured using one-way ANOVA. The bars and error bars represent mean ± SD. qPCR, quantitative PCR; OA, oleic acid.

To determine whether loss of *APOB* gene might cause steatosis, we measured TG levels in Ako cells by Oil Red O staining and after total lipid extraction. We did not observe significant differences in the cellular lipid stain in the Ako cells as compared to the control Huh-7 cells (Fig. 4A, B). Moreover, TG quantification after isopropanol extraction showed similar TG levels in control and Ako cells (Fig. 4C). Thus, these Ako cells do not accumulate higher amounts of TG under these culture conditions.

We next checked the mRNA expression of genes in unfolded protein response such as activating transcription factor 4, activating transcription factor 6, Binding immunoglobulin protein and X-box binding protein 1 (XBP1). For XBP1, we measured the mRNA levels of unspliced (XBP1-u) and spliced (XBP1-s) isoforms (33). We did not observe significant difference in the mRNA expression of genes involved in unfolded protein response between Ako and Huh-7 cells (Fig. 4D). Furthermore, Ako and Huh-7 cells were grown and maintained in the presence of 0.2 mM OA and mRNA levels of unfolded protein response genes were quantified. In the presence of OA as well, we did not observe significant differences in the mRNA levels of these genes between the two groups (Fig. 4E). Thus, *APOB* gene deficiency does not appear to cause unfolded protein response under these conditions.

### Global transcriptome changes due to *APOB* gene ablation

**Figure 5:**
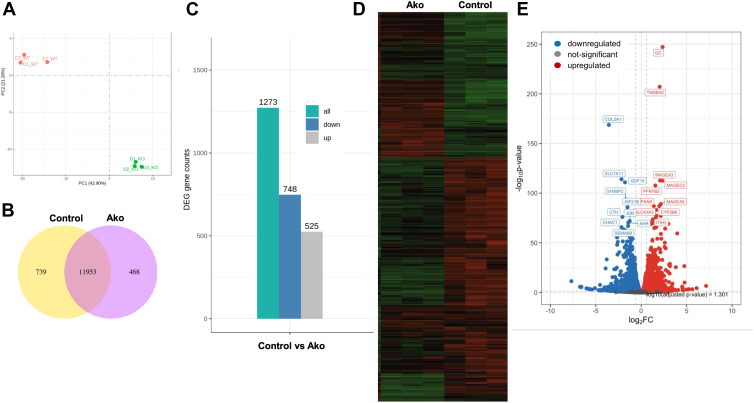
*APOB* gene ablation leads to differential gene expression in Ako cells. A: We performed principal component analyses on the gene expression values based on fragments per kilo base of transcript per million base pairs sequenced (FPKM). B: The Venn diagram shows the unique expression of genes in Huh-7 and Ako cells. C: Data from differential gene expression analysis are shown as histograms. The numbers on the columns indicate the number of differentially expressed genes. D: For cluster analysis of differentially expressed genes, we used mainstream hierarchical clustering using FPKM values of genes. The genes with similar expression patterns in the heat map are clustered together. The color in each grid reflects homogenized expression data for horizontal comparison only. E: Volcano plot of differentially expressed genes. The *x*-axis shows the fold change in gene expression between different samples, and the *y*-axis shows the statistical significance of the differences. Red dots represent upregulated genes and blue dots represent downregulated genes.

To study global effects of *APOB* gene ablation on transcriptome, we performed RNA-Seq analysis in control and Ako-3 cells in triplicate. To evaluate intra and intergroup differences, we performed principal component analysis on the gene expression values of all samples. Samples within WT and Ako-3 triplicates were clustered together, whereas two groups segregated into two distinct clusters (Fig. 5A). These studies indicated that expression profiles within groups were similar and there were significant differences among the two groups of cells and prompted us to compare differences between them. The coexpression Venn diagram showed that 11,953 genes were expressed in both control and Ako cells, 739 genes were only expressed in control, while 468 genes were uniquely expressed in Ako cells only (Fig. 5B). Differential gene expression analysis (log_2_(fold change) ≥1 and padj ≤0.05) between Ako and control cells revealed that 1273 genes were differentially expressed. Within these changed transcripts 525 and 748 mRNA levels were upregulated and downregulated, respectively, in Ako cells (Fig. 5C). Similarly, heat map showed intragroup similarities and intergroup differences in gene expression patterns (Fig. 5D). To get an overall distribution of differentially expressed genes, we displayed data as Volcano plots (Fig. 5E) and identified top 20 differentially expressed transcripts. Few characteristic features of these genes are in Supplemental Table 1. The top upregulated genes in the Ako-3 cells were *GC* that codes for VDBP and *TMSB4X* which codes for thymosin beta 4 X linked. The most downregulated gene was *COL2A1* which codes for the collagen type II alpha 1 chain. ApoB mRNA levels were reduced by ∼60% in the transcriptome analysis (not shown) like our qRT-PCR results (Fig. 1C). Next, we performed enrichment analysis of the differentially expressed genes and identified different affected processes and pathways using ClusterProfiler. First, we performed Gene Ontology (GO) to identify downregulated and upregulated biological processes (BPs), cellular components (CCs), and molecular functions (MFs). Highly downregulated BPs were related to receptor signaling and inorganic compound detoxification (Supplementary Fig. 3A). Surprisingly, upregulated BPs were related to DNA replication and immune response (Supplementary Fig. 3B). Analyses of CCs revealed that extracellular matrix–related components were highly downregulated in Ako cells (Supplementary Fig. 3A), whereas vesicular components were upregulated (Supplementary Fig. 3B). Analyses of MFs showed that receptor ligand activities were significantly downregulated in Ako cells (Supplementary Fig. 3A), whereas MFs related to peptidases and endopeptidases were upregulated (Supplementary Fig. 3B). It is likely that these enzymes might be involved in the quality control of apoB-containing lipoprotein assembly and secretion as it is well established that intracellular proteolysis is a major mechanism controlling apoB secretion.

**Figure 6:**
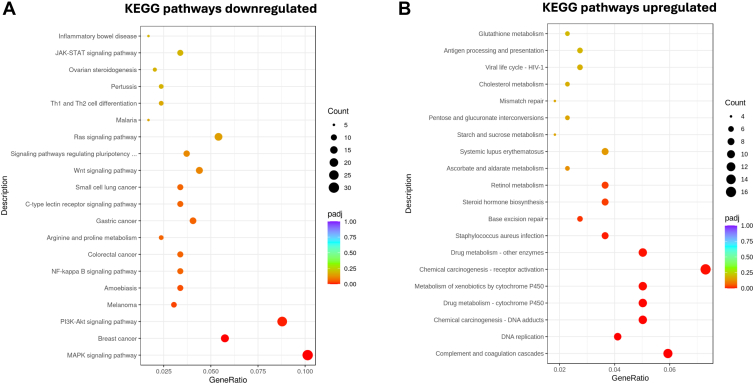
KEGG pathway analyses of differentially expressed genes in Ako-3 cells. Differential gene enrichment analysis data were used to find out biological functions or pathways that were significantly affected by *APOB* gene ablation. We used clusterProfiler software for different enrichment analysis. A and B: Kyoto Encyclopedia of Genes and Genomes (KEGG) was used to identify significantly enriched metabolic pathways and signal transduction pathways that were downregulated (A) or upregulated (B).

**Figure 7:**
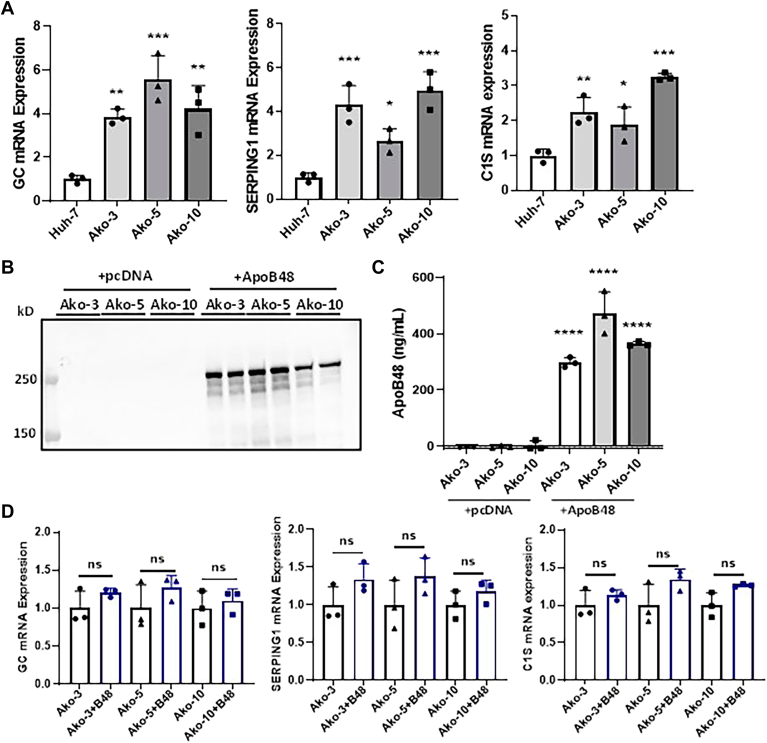
ApoB48 expression has no effect on the expression of highly upregulated genes in Ako cells. A: Validation of transcriptome results with qPCR. Ako and Huh-7 cells were plated in 6-well plates at a density of 2 × 10^5^ cells per well. After 48 h, total RNA from Huh-7 and Ako cells was isolated by Trizol method and used to measure relative mRNA levels. The bars and error bars represent mean ± SD. To calculate the significance, one-way ANOVA was used. ∗*P* < 0.05, ∗∗*P* < 0.01, and ∗∗∗*P* < 0.001. B: Ako clones were transfected with 3 μg plasmid expressing either apoB48 or pcDNA empty vector control. After 48 h, media was changed and apoB48 expression was studied in the overnight condition media by Western blot. C: The overnight media collected from Ako cells was also used to measure apoB48 by ELISA. Comparison between pcDNA and apoB48 expressing cells; ∗∗∗∗, *P* < 0.0001, Student *t* test. D: Total RNA was collected form the transfected Ako cells using Trizol method and qPCR was performed to measure relative mRNA expression of differentially expressed genes. The bars and error bars represent mean ± SD. Multiple *t* test was used to compare statistical significance between two groups. ns, not significant; qPCR, quantitative PCR.

Next, we performed Kyoto Encyclopedia of Genes and Genomes (KEGG) enrichment analysis to identify biological pathways predominantly affected by *APOB* gene deletion. MAPK signaling pathways related to breast cancer and PI3K-AKT signaling were the most downregulated pathway (Fig. 6A). The most upregulated pathways were DNA replication, complement, and coagulation (Fig. 6B). Some of the most significantly changed KEGG pathways and associated genes in these pathways are summarized in Supplemental Table 2. Reactome enrichment pathway analyses revealed that Ako cells had significantly downregulated response to metal ions and interleukin signaling (Supplementary Fig. 4A). Consistent with other pathway analyses, the highly upregulated reactomes were related to DNA unwinding, elongation and replication (Supplementary Fig. 4B). Next, we performed qRT-PCR for a few candidate genes to confirm these changes. Transcriptome data identified *GC* mRNA as the most upregulated transcript (Fig. 5E). This was confirmed by qRT-PCR (Fig. 7A). Similarly, mRNA levels of serine protease inhibitor 1 (*SERPING1*) and complement component 1s in the complement/coagulation pathway were increased in Ako cells compared to control cells (Fig. 7A).

The transgenic expression of apoB48 leads to secretion of apoB48 lipoproteins in the Ako cells. Therefore, we next asked whether the expression of apoB48 would revert the gene expression changes observed in Ako cells. To answer this, we transfected the Ako cells with plasmid expressing either apoB48 or pcDNA and then studied the mRNA levels of *GC*, *SERPING1,* and complement component 1s. We did not observe significant difference in the mRNA expression of these genes after the expression of apoB48 (Fig. 7B, D) suggesting that upregulation of the expression of these genes might be a long-term adaptive response.

We next identified the protein network clusters affected by apoB deficiency using the STRING analysis database version 12.0. The STRING analysis identified around 150 clusters based on the differentially expressed genes in Ako cells (Supplementary Fig. 5A). Some of the identified dense clusters belonged to pathways such as DNA replication, metabolism of xenobiotics, cell cycle regulation, and complement activation (Supplementary Fig. 5B–E). Thus, transcriptome analysis revealed that several pathways were affected by *APOB* gene deletion.

### The secretory protein profile of Ako cells differs significantly from the control Huh-7 cells

**Figure 8:**
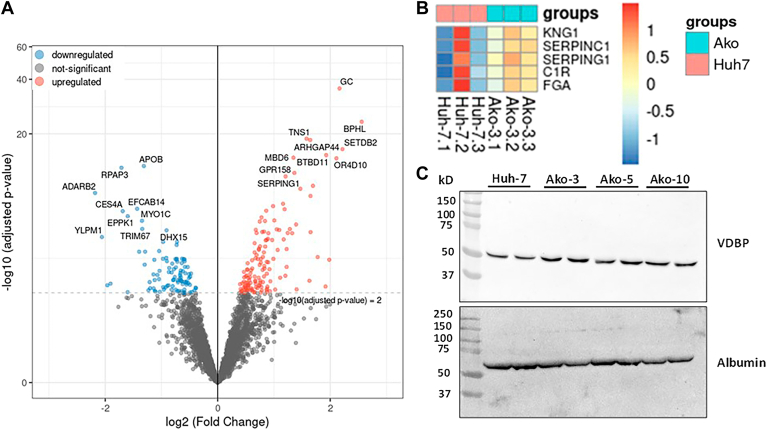
Validation of proteome results with Western blot analysis. A: Conditioned media from three different flasks of control and Ako-3 cells were separately concentrated using Centricon-10 filters. The samples were subjected to proteomic analysis. Cluster analysis of differentially expressed proteins was performed. The volcano plots identified VBDP (gene *GC*) as the most upregulated protein in the Ako cell media and apoB was the most downregulated protein. B: Heat map for differentially expressed proteins in complement and coagulation pathways in the media of Ako cells. C: For Western blot analysis, confluent cell cultures were incubated overnight in serum-free media. The conditioned media was used to detect VDBP protein. VDBP, vitamin D binding protein.

To study the effect of apoB loss on the total secretory protein profile, we performed proteomic analysis in the overnight serum-free conditioned media obtained from Ako and Huh-7 cells. Volcano plot of differentially expressed proteins revealed that the most downregulated protein in Ako media was apoB (Fig. 8A). This was expected and was consistent with our data in Figure 1. The KEGG enrichment analysis from the proteomics data identified complement and coagulation pathway to be significantly upregulated in the Ako cells (Fig. 8B). The highly upregulated protein was VDBP (Fig. 8A). This was confirmed by Western blot analysis (Fig. 8C). The top 20 most significantly changed proteins from proteomic analysis are in Supplemental Table 3. We also performed STRING pathway analysis to identify protein networks affected in these cells (Supplementary Fig. 6A). After clustering, we identified four major clusters with more than 10 or more proteins in each network. The major clusters identified based on proteomics data were related to plasma lipoprotein assembly, remodeling and clearance, complement and coagulation cascade, and spliceosome and RNA processing (Supplementary Fig. 6B–E). Thus, secretome of the hepatoma cells is affected by *APOB* gene ablation.

### Total transcriptome changes in the *MTTP* knockout cells

Since MTP and apoB both are critical players in the assembly and secretion of apoB-containing lipoproteins, we performed transcriptome analysis in our previously generated *MTTP* deficient Huh-7 cells (Mko) (12). The principal component analysis showed two distinct clusters for control Huh-7 and Mko cells (Supplementary Fig. 7A). The coexpression Venn diagram displayed 11855 genes to be common and expressed in both control and Mko cells, while 837 and 476 genes were uniquely expressed by Huh-7 and Mko cells, respectively. Furthermore, differential gene expression analysis showed differential expression of 1679 genes, where 1089 were significantly downregulated in Mko and 590 genes were upregulated in Mko cells as compared to control Huh-7 (Supplementary Fig. 7B, C). The overall transcriptome difference between two groups was displayed as heat map, where the two groups showed significantly distinct gene expression profile (Supplementary Fig. 7D). The KEGG enrichment analysis showed downregulation of pathways related to breast cancer in Mko-3 cells, like Ako-3 cells (Supplementary Fig. 8A). The common upregulated pathways among Ako-3 and Mko-3 cells were found to be DNA replication, base excision repair, and steroid hormone biosynthesis (Supplementary Fig. 8B). We also performed GO in Mko-3 cells to identify downregulated and upregulated BPs, CCs, and MFs. The GO analysis showed significant downregulation of BPs related to fatty acid metabolism in Mko-3 cells, and like Ako cells the downregulated CCs were extracellular matrix (Supplementary Fig. 9A). The upregulated pathways in GO analysis were related to DNA replication and cell cycle progression (Supplementary Fig. 9B). Thus, similar pathways are affected by both *MTTP* and *APOB* gene ablations.

## Discussion

We report successful establishment of Huh-7 cell clones deficient in the expression of *APOB* gene. These clones did not secrete apoB despite normal MTP expression and activity. Furthermore, transgenic expression of apoB48 led to its secretion as a lipoprotein particle. Thus, these cells retain all the machinery needed for the assembly and secretion of apoB-containing lipoproteins, except for the *APOB* gene transcript and protein expression. Therefore, these clones can be used to study the effects of different known and novel variants on apoB function.

The Ako cells have normal MTP activity. Since apoB is known to be the major substrate for MTP, it is intriguing that there were no changes in MTP expression. It is possible that MTP has other functions in liver cells that we are unaware of. However, we know that CD1 proteins are substrates for MTP in NKT cells (34, 35). We have previously shown that MTP in the adipose tissue interacts with ATGL and inhibits its activity (36). A recent study showed that MTP interacts with Pla2g12b in liver cells (37). In earlier studies, we noted that *MTTP* gene ablation had no effect on apoB expression (12). Thus, these two essential proteins in lipoprotein assembly do not affect each other’s expression.

Transgenic expression of apoB48 resulted in secretion of apoB in media as a smaller LDL particle as than Huh-7 cells (Fig. 3C). The smaller LDL is because of the smaller apoB48 peptide length than apoB100 in Huh-7 cells. Zemin Yao *et al.* (38) have shown that size of lipoproteins secreted by hepatoma cells depends on the length of the apoB peptide. We have also observed similar relationship between the elution profiles of apoB48- and apoB100-containing lipoproteins (19). Therefore, the primordial particles assembled using apoB48 and apoB100 peptides show different flotation properties and elution profiles.

We did not see significant accumulation of lipids in Ako cells suggesting that loss of apoB does not lead to accumulation of TGs under these culture conditions. These findings agree with previous studies where the knock down of apoB mRNA has been shown to reduce plasma cholesterol levels without causing significant steatosis in mice (39). Later Conlon *et al.* also showed that loss of apoB reduced VLDL secretion and plasma cholesterol without causing steatosis in mice (40) They suggested that ER stress–induced ER-phagy may avoid hepatic lipid accumulation. Total transcriptomics and qRT-PCR analyses did not show upregulation of pathways related to ER autophagy and unfolded protein response. Therefore, it remains unexplained how these cells escape lipid accumulation without triggering unfolded protein response.

ApoB is considered a scaffolding protein for the assembly and secretion of lipoproteins. Therefore, we had anticipated very little effect on different cellular pathways after gene ablation. To our surprise, RNA-Seq analysis identified DNA replication, complement, and coagulation pathways as the most upregulated pathways. Lipids are known to maintain genome integrity by maintaining cellular levels of histones and it has been shown that lipid derived acetyl CoA participates in histone acetylation, thereby regulating gene expression (41). However, Ako cells did not show significant difference in growth rate as compared to the control Huh-7 cells. Perhaps, these cells are primed for replication, but an additional signal is required to trigger proliferation. Similar to Ako cells, we have also reported that loss of *MTTP* in Huh-7 cells has no significant effect on cell growth (12).

Lipoproteins are also known to carry proteins which are not involved in lipid metabolism, such as those involved in the blood coagulation cascade (42, 43). Hypercholesterolemic patients undergoing LDL apheresis have reduced levels of blood clotting–related proteins which are carried on apoB lipoproteins (44). Recently, the interaction between coagulation pathway and apoB lipoprotein has been studied where the tissue plasminogen activator has been shown to directly interact with apoB and inhibit its interaction with MTP in the ER. This blocks the apoB lipidation resulting in lower levels of apoB-containing lipoproteins in the plasma (45). Our transcriptome and proteome studies identified coagulation and complement cascades as one of the most upregulated pathways in the Ako cells. The RNA-Seq analysis identified a total of 13 genes to be differentially upregulated in this pathway, while secretome analysis identified increased secretion of kininogen, fibrinogen alpha chain, C1r, Serping*,* and anti-thrombin III (SERPINC1) in Ako cells. These studies for the first time highlight the presence of transcriptional control mechanisms that are linked to apoB expression.

The fat-soluble vitamins require lipoproteins for their transport to peripheral tissues and loss of apoB-containing lipoproteins in plasma is associated with poor absorption of vitamins A, E, D, and K (9, 10). Besides lipoproteins, fat-soluble vitamins are carried in plasma bound to specific proteins. There is very little information about relationships between apoB-containing lipoproteins and fat-soluble vitamin transport proteins. It is possible that VDBP has been increased to mobilize vitamin D metabolites in the absence of lipoprotein assembly. VDBP delivers dietary and endogenously produced vitamin D metabolite to peripheral tissues and also plays an important role in actin scavenging toward off inflammatory and immune response (46). VDBP binds the active metabolite 25 hydroxyvitamin D (25(OH)D) with highest affinity and maintains the circulating levels of 25(OH)D. We are unaware of studies reporting increased expression of apoB in the settings of reduced VBDP expression.

This study identified the effect of apoB deficiency on several unanticipated cellular pathways. Therefore, we also studied the global gene expression changes in *MTTP*-deficient Huh-7 cells (12). We found upregulation of common pathways such as DNA replication and steroid hormone synthesis in both Ako and Mko cells. In the present study, we only looked at overall changes in the cells due to *APOB* and *MTTP* loss. Another caveat of this study is that it only shows correlative changes associated with apoB gene deficiency. Mechanistic studies explaining how apoB deficiency causes changes in these pathways need to be performed. Furthermore, these observations need further validation in animal models.

In short, our studies establish new hepatoma cell lines that are deficient in apoB expression and retain other machinery needed for lipoprotein assembly and secretion. These studies uncovered unanticipated relationships between apoB and various cellular pathways such as DNA replication, complement/coagulation pathways, and MAPK signaling. These cells may be useful in studying structure-function analysis of apoB variants and to study their effects on other cellular pathways.

## Data availability

All the data are in the article. RNA-Seq data have been deposited in GEO.

## Supplemental data

This article contains supplemental data.

## Conflict of interest

The authors declare that they have no conflicts of interest with the contents of this article.

## Supplementary data

Supplementary Figures

Supplementray Tables
