# Supplementary material for: Human hepatoma Huh-7 cell culture models deficient in apolipoprotein B secretion
Source: J Lipid Res. 2025 Jul 23;66(8):100867. doi: 10.1016/j.jlr.2025.100867 (PMC12396022; doi:10.1016/j.jlr.2025.100867)
Supplement: Supplementary Figures [file mmc1.pdf]

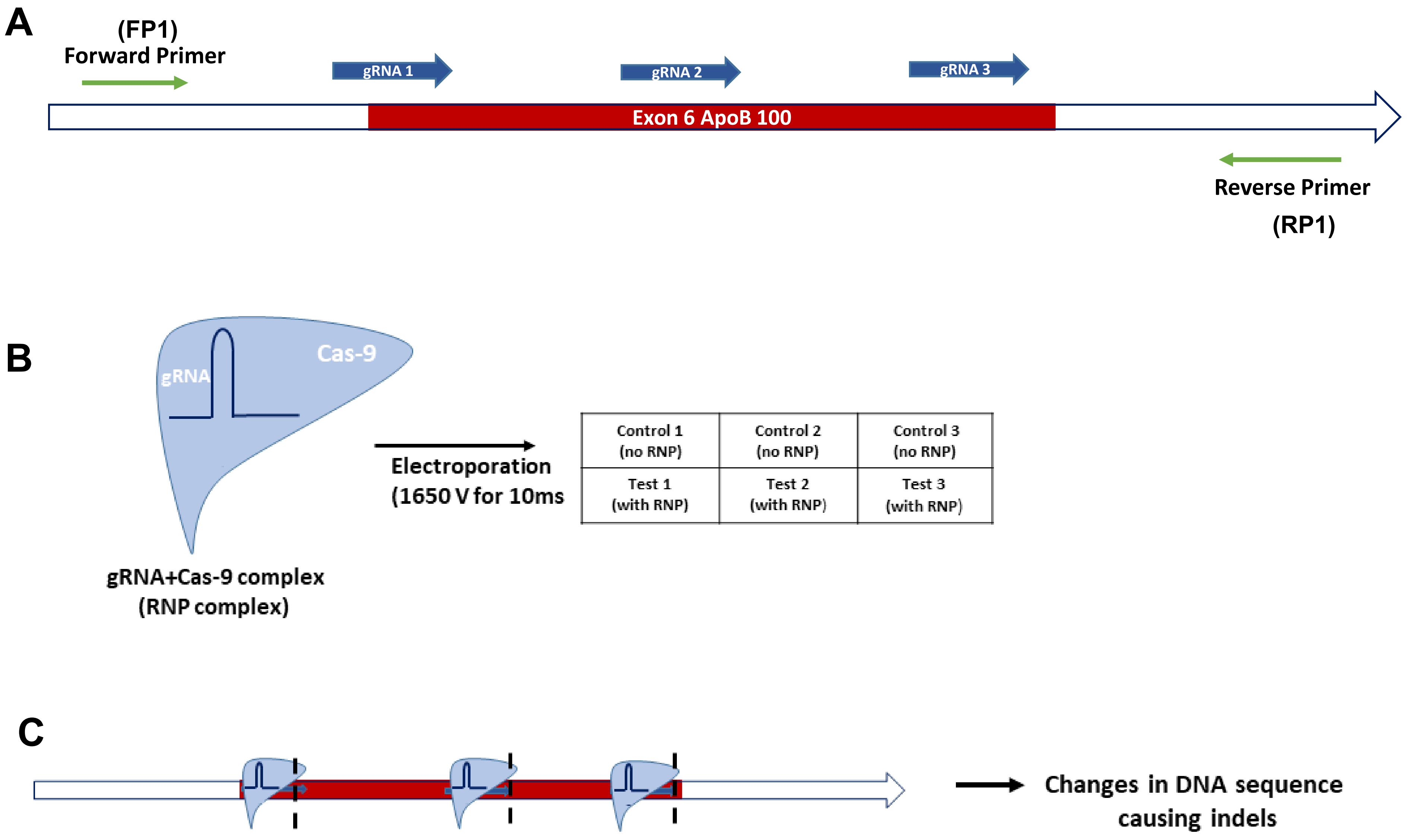
A
(FP1)
Forward Primer
gRNA 1
gRNA 2
gRNA 3
Exon 6 ApoB 100
Reverse Primer
(RP1)
B
gRNA
Cas-9
gRNA+Cas-9 complex
(RNP complex)
Electroporation
(1650 V for 10ms
Control 1 (no RNP)
Control 2 (no RNP)
Control 3 (no RNP)
Test 1 (with RNP)
Test 2 (with RNP)
Test 3 (with RNP)
C
Changes in DNA sequence
causing indels

Supplementary Figure 1: sgRNA mediated deletion of *APOB* gene.

(A) Three gRNAs (represented by blue arrows) targeting 160 bp region within the exon six of *APOB* gene were selected based on minimal off target effects. Forward and reverse primers (green arrows) were designed for the PCR amplification of the targeted region and for sequencing of the PCR products.

(B) To form the RNP complex, three gRNAs (30 µM) and Cas9 nuclease (20 µM) were mixed in microcentrifuge tubes and incubated at room temperature for 15 min. The RNA-protein complexes were then electroporated into the Huh-7 cells. For control, Huh-7 cells were treated similarly without any RNP complexes. Cells transfected with or without RNP complexes were placed in 6-well plates containing prewarmed media and allowed to grow at 37°C for one week. Media was changed every 48 h and cells were monitored for growth by observing under microscope.

(C) After entering the cell, the gRNA recognizes and binds to homologous sequence within the targeted region and directs the Cas-9 nuclease to carry out double stranded breaks in the DNA. Following this, DNA undergoes error prone non-homologous end joining (NHEJ) which results in the random mutations resulting in frame shifts and indels in the DNA sequence.

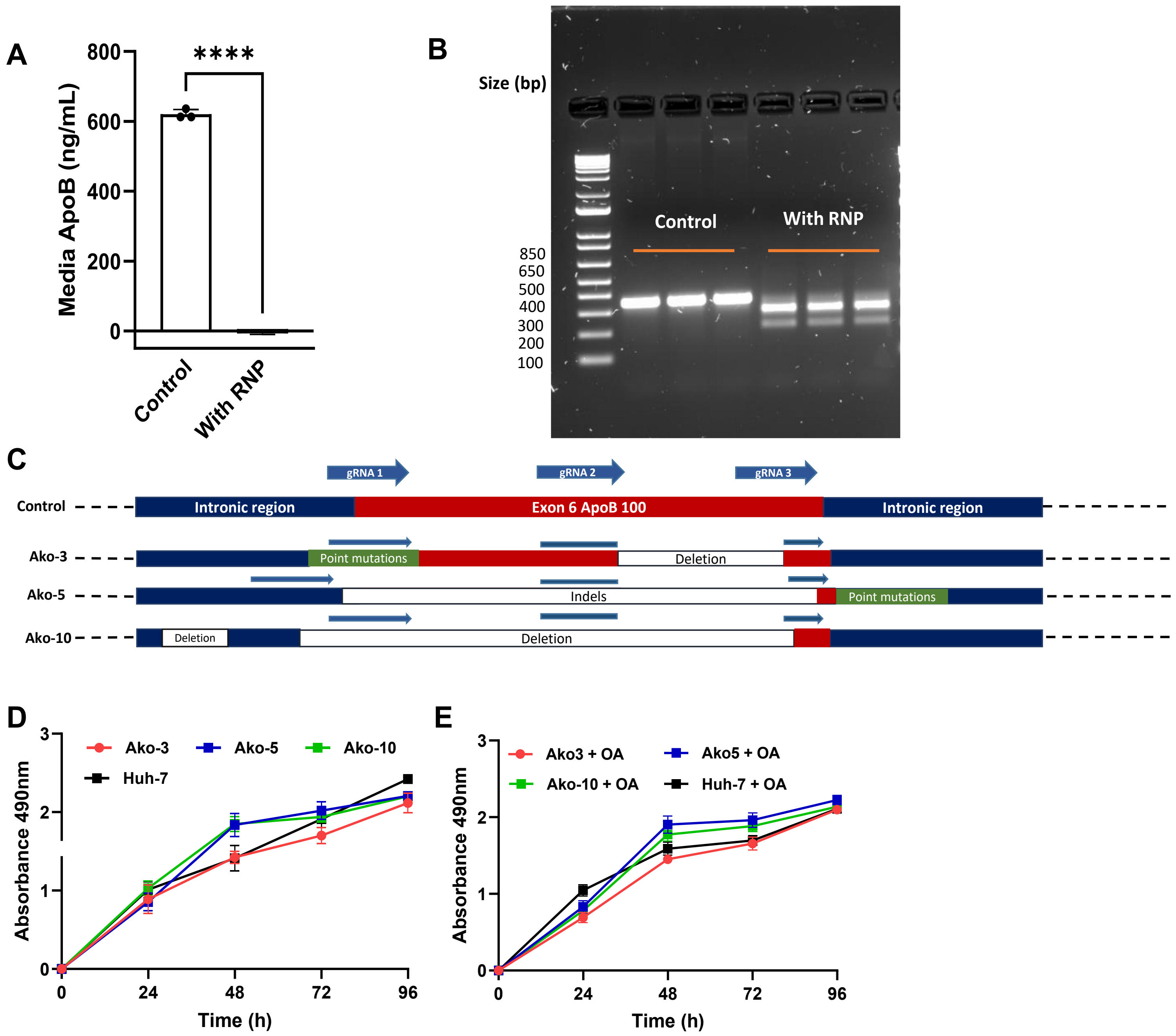
A
****
Media ApoB (ng/mL)
800
600
400
200
0
Control
With RNP
B
Size (bp)
850
650
500
400
300
200
100
Control
With RNP
C
gRNA 1
gRNA 2
gRNA 3
Control
Intronic region
Exon 6 ApoB 100
Intronic region
Ako-3
Point mutations
Deletion
Ako-5
Indels
Point mutations
Ako-10
Deletion
Deletion
D
Ako-3
Ako-5
Ako-10
Huh-7
Absorbance 490nm
Time (h)
0
24
48
72
96
E
Ako3 + OA
Ako5 + OA
Ako-10 + OA
Huh-7 + OA
Absorbance 490nm
Time (h)

Supplementary Figure 2: Primary screening for gene ablation and possible indels.
(A) The Huh-7 cells transfected with and without RNP complexes were grown in 6-well plates until they were 80-90% confluent with media changed at every 48 h. After 1 week, overnight conditioned media was collected from the 6-well plates and apoB was measured by ELISA.
(B) Genomic DNA was isolated from the cells, the targeted region within the exon 6 of *APOB* gene was PCR amplified and resolved on 2% agarose gel. The first three lanes represent control cells which did not receive RNP complexes and later three lanes represent the test samples transfected with the RNP complexes.
(C) The PCR products from Fig 1B were purified and sequenced using forward and reverse primers to look at deletions and indels. The sequencing data were aligned with the reference sequence using SnapGene and changes in the DNA sequence were identified in each Ako clone.
(D) Ako and Huh-7 cells were seeded in 96 well plates at a density of 2000 cells per well and cell proliferation was monitored by adding assay reagent after 24 h, 48 h, 72 h and 96 h and measuring absorbance at 490 nm.
(E) Ako and Huh-7 cells were grown and maintained in 96 well plates in DMEM media supplemented with 0.2 mM oleic and 1.5% BSA. Cell proliferation was studied by adding assay reagent at regular intervals of 24 h and absorbance was measured at 490 nm.

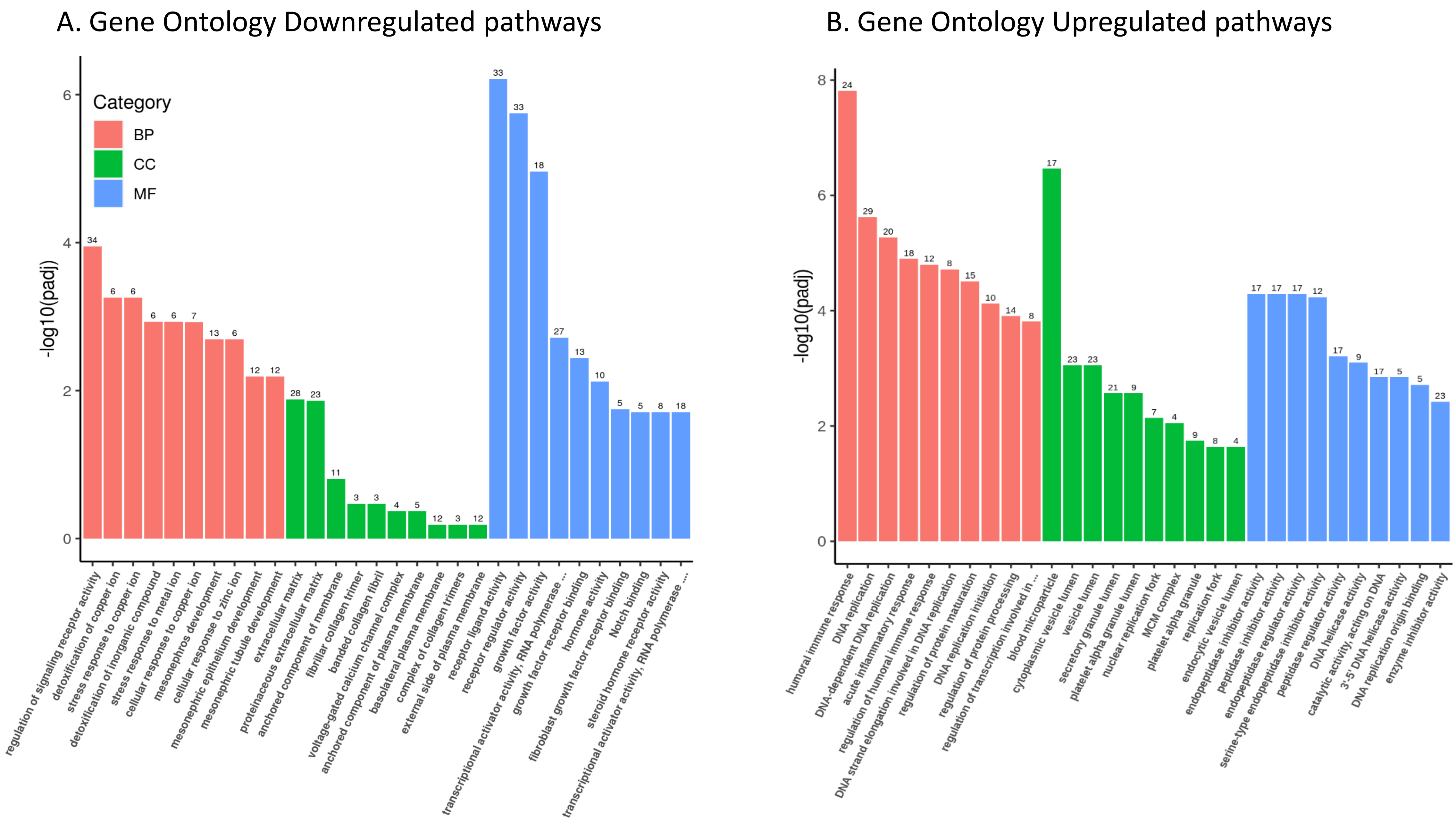
A. Gene Ontology Downregulated pathways
B. Gene Ontology Upregulated pathways
Category
BP
CC
MF
-log10(padj)
regulation of signaling receptor activity
detoxification of copper ion
stress response to copper ion
detoxification of inorganic compound
stress response to metal ion
cellular response to copper ion
mesonephros development
cellular response to zinc ion
mesonephric epithelium development
mesonephric tubule development
extracellular matrix
proteinaceous extracellular matrix
anchored component of membrane
fibrillar collagen trimer
banded collagen fibril
voltage-gated calcium channel complex
anchored component of plasma membrane
basolateral plasma membrane
complex of collagen trimers
external side of plasma membrane
receptor ligand activity
receptor regulator activity
growth factor activity
transcriptional activator activity, RNA polymerase ...
growth factor receptor binding
hormone activity
fibroblast growth factor receptor binding
Notch binding
steroid hormone receptor activity
transcriptional activator activity, RNA polymerase ...
humoral immune response
DNA replication
DNA-dependent DNA replication
acute inflammatory response
regulation of humoral immune response
DNA strand elongation involved in DNA replication
regulation of protein maturation
DNA replication initiation
regulation of protein processing
regulation of transcription involved in ...
blood microparticle
cytoplasmic vesicle lumen
vesicle lumen
secretory granule lumen
platelet alpha granule lumen
nuclear replication fork
MCM complex
platelet alpha granule
replication fork
endocytic vesicle lumen
endopeptidase inhibitor activity
peptidase inhibitor activity
endopeptidase regulator activity
serine-type endopeptidase inhibitor activity
peptidase regulator activity
DNA helicase activity
catalytic activity, acting on DNA
3'-5' DNA helicase activity
DNA replication origin binding
enzyme inhibitor activity

Supplementary Figure 3: Human Gene Ontology was used to identify differentially regulated pathways in Ako cells
(A-B) Gene Ontology (GO) presentation includes three main branches: biological processes (BP), cellular component (CC), and molecular function (MF). Down regulated (A) and up-regulated (B) GO terms are plotted separately.

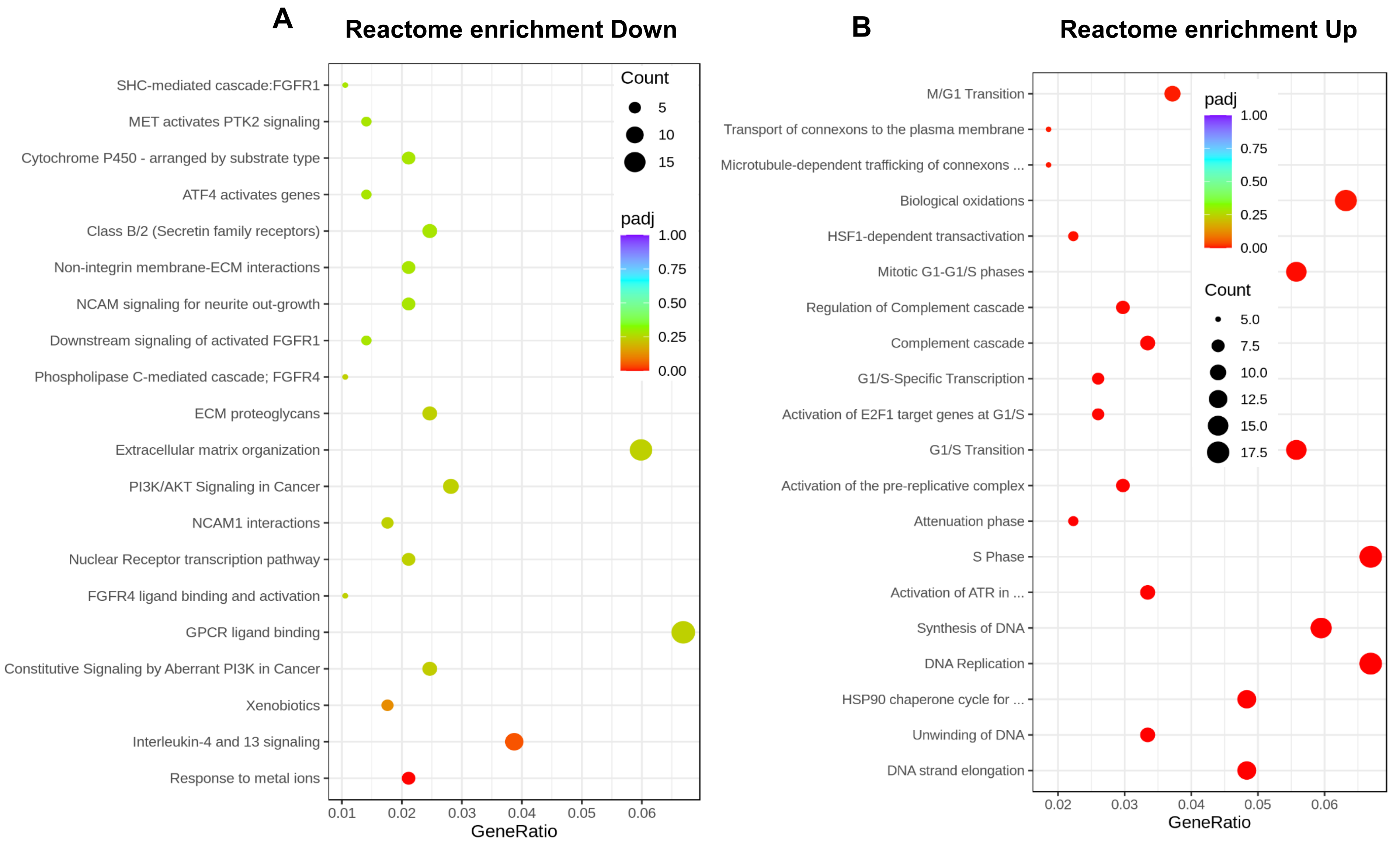

Supplementary Figure 4: Reactome enrichment pathway analyses

(A-B) Reactome enrichment pathway analysis was done to identify significantly down (A) and upregulated (B) pathways in Ako cells.

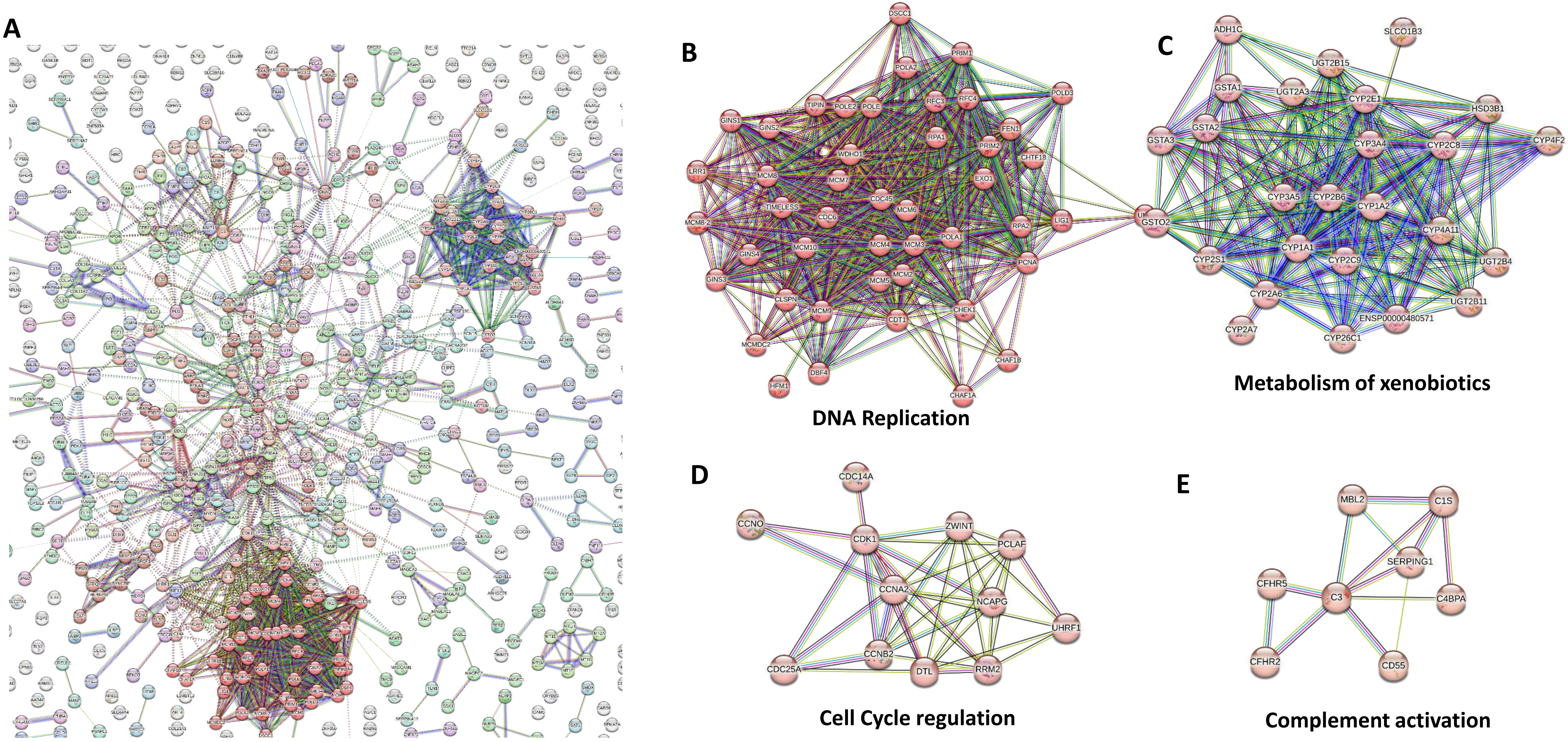
A
B
DSCC1
PRIM1
POLA2
POLD3
TIPIN
POLE2
POLE
RFC3
RFC4
GINS1
GINS2
RPA1
FEN1
PRIM2
CHTF18
LRR1
WDHD1
MCM8
MCM7
EXO1
CDC45
TIMELESS
MCM6
CDC6
MCM8-2
LIG1
RPA2
POLA1
MCM4
MCM3
GINS4
MCM10
PCNA
GINS3
MCM2
MCM5
CLSPN
CHEK1
MCM9
CDT1
MCMDC2
CHAF1B
DBF4
HFM1
CHAF1A
DNA Replication
C
ADH1C
SLCO1B3
UGT2B15
GSTA1
UGT2A3
CYP2E1
HSD3B1
GSTA2
GSTA3
CYP3A4
CYP2C8
CYP4F2
GSTO2
CYP3A5
CYP2B6
CYP1A2
CYP4A11
CYP1A1
CYP2S1
CYP2C9
UGT2B4
CYP2A6
UGT2B11
ENSP00000480571
CYP2A7
CYP26C1
Metabolism of xenobiotics
D
CDC14A
CCNO
CDK1
ZWINT
PCLAF
CCNA2
NCAPG
UHRF1
CCNB2
CDC25A
DTL
RRM2
Cell Cycle regulation
E
MBL2
C1S
SERPING1
CFHR5
C3
C4BPA
CFHR2
CD55
Complement activation

Supplementary Figure 5: STRING pathway analysis to identify apoB related protein network based on total transcriptomic data in wildtype and Ako cells.

(A) The apoB related protein networks were displayed and clustered to identify associated pathways.

(B-E) Some of the identified clusters belonged to pathways such as DNA replication, metabolism of xenobiotics, cell cycle regulation, and complement activation.

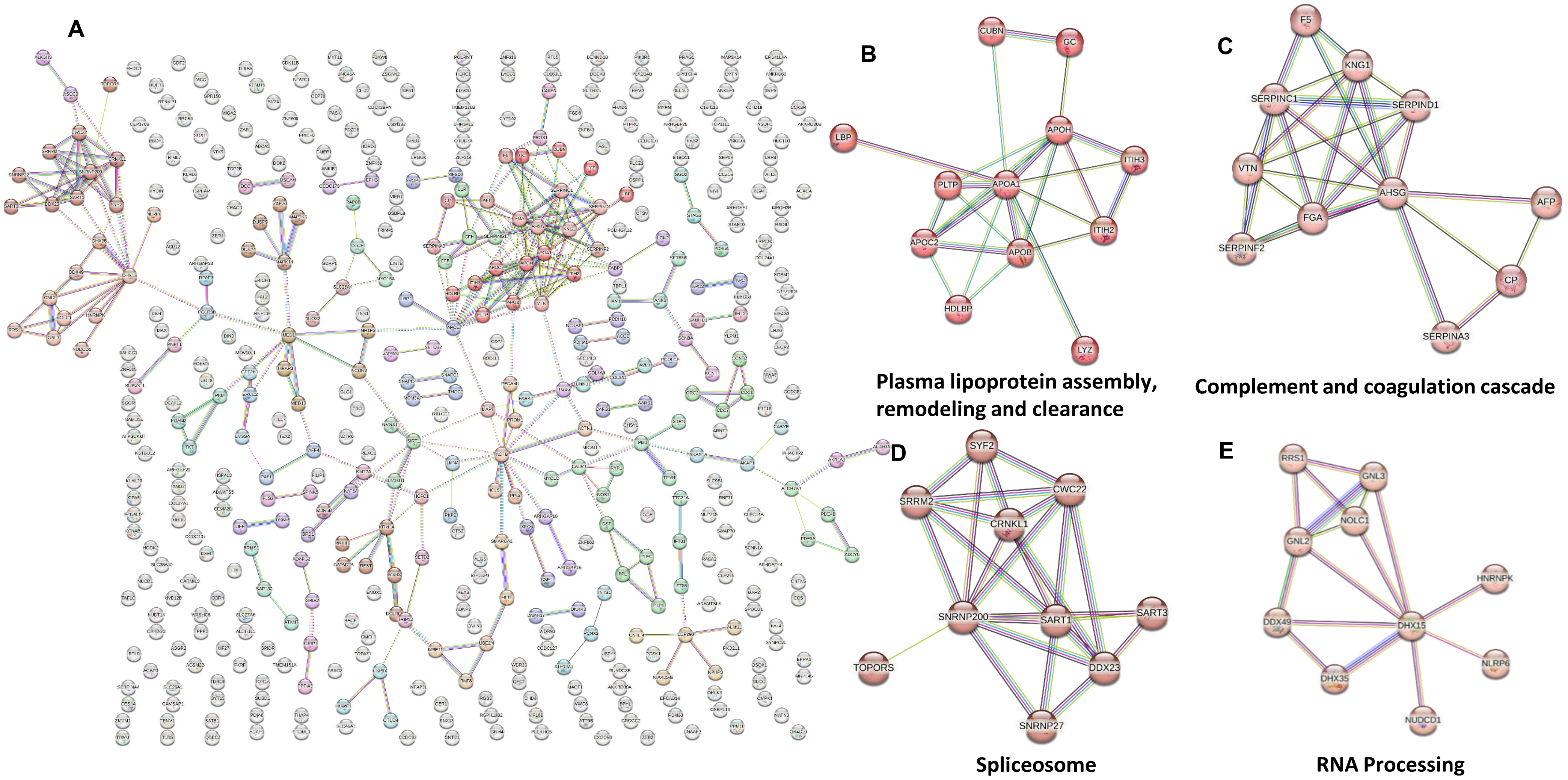

Supplementary Figure 6: STRING pathway analysis to identify apoB related protein network based on proteomics data from the conditioned media obtained from wildtype and Ako Huh-7 cells.

(A) The APOB related protein networks were displayed and clustered to identify associated pathways.

(B-E) Clusters with ten or more proteins are displayed.

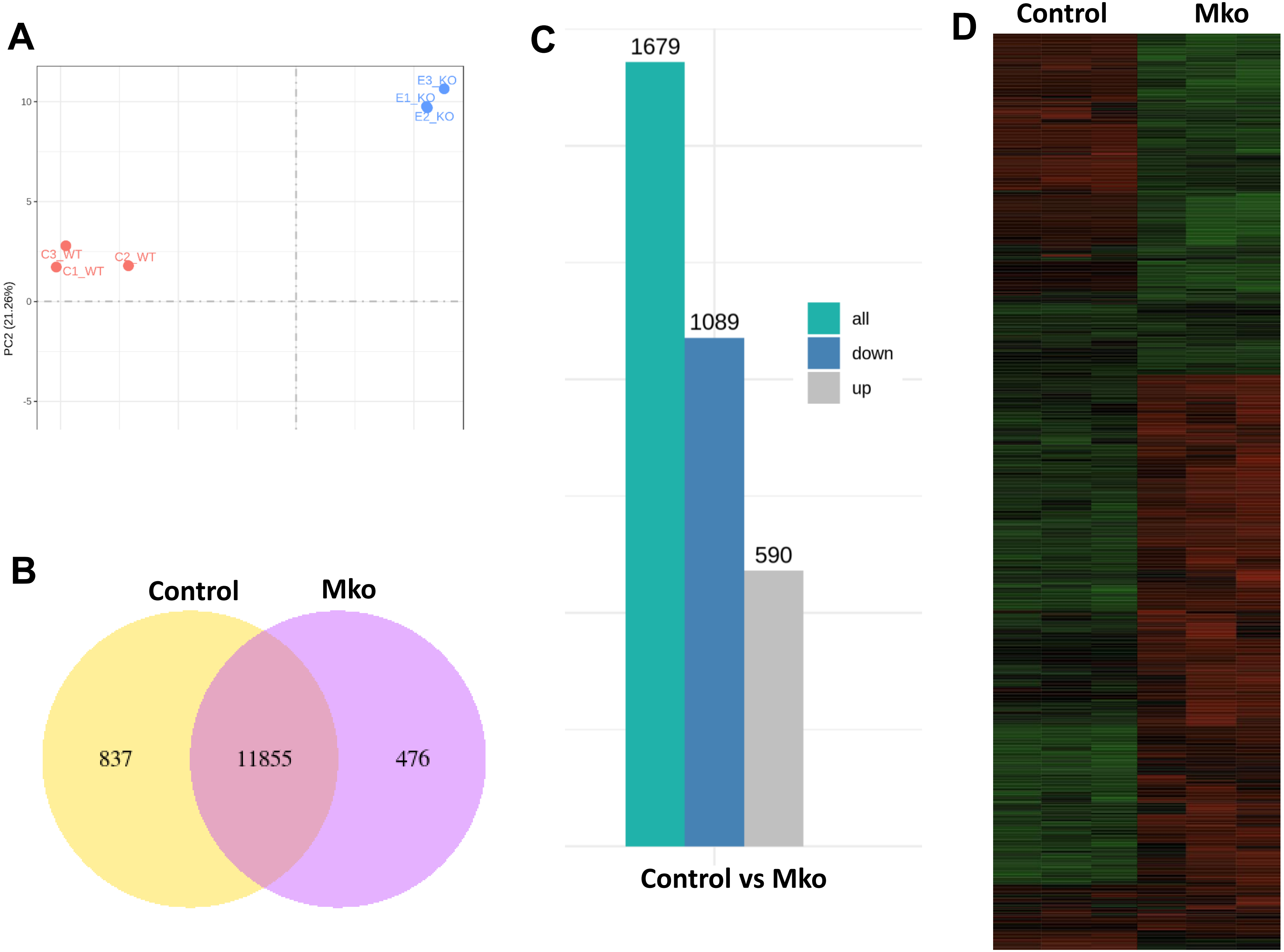
A
10
5
0
-5
PC2 (21.26%)
E3_KO
E1_KO
E2_KO
C3_WT
C1_WT
C2_WT
B
Control
Mko
837
11855
476
C
1679
1089
590
all
down
up
Control vs Mko
D
Control
Mko

Supplementary Figure 7: Total transcriptomics analysis in *MTTP* gene ablated (Mko-3) Huh-7 cells

(A) Principal component analysis shows two distinct clusters for control Huh-7 and Mko cells.

(B) Co-expression Venn diagram showing unique expression of gene in two groups

(C) Differentially expressed genes based on ($\log_2$(fold change $\geq$ 1 and padj $\leq$ 0.05) are displayed as bar graph.

(D) Intra group similarities and inter group differences in gene expression between control and Mko cells are displayed as heat map.

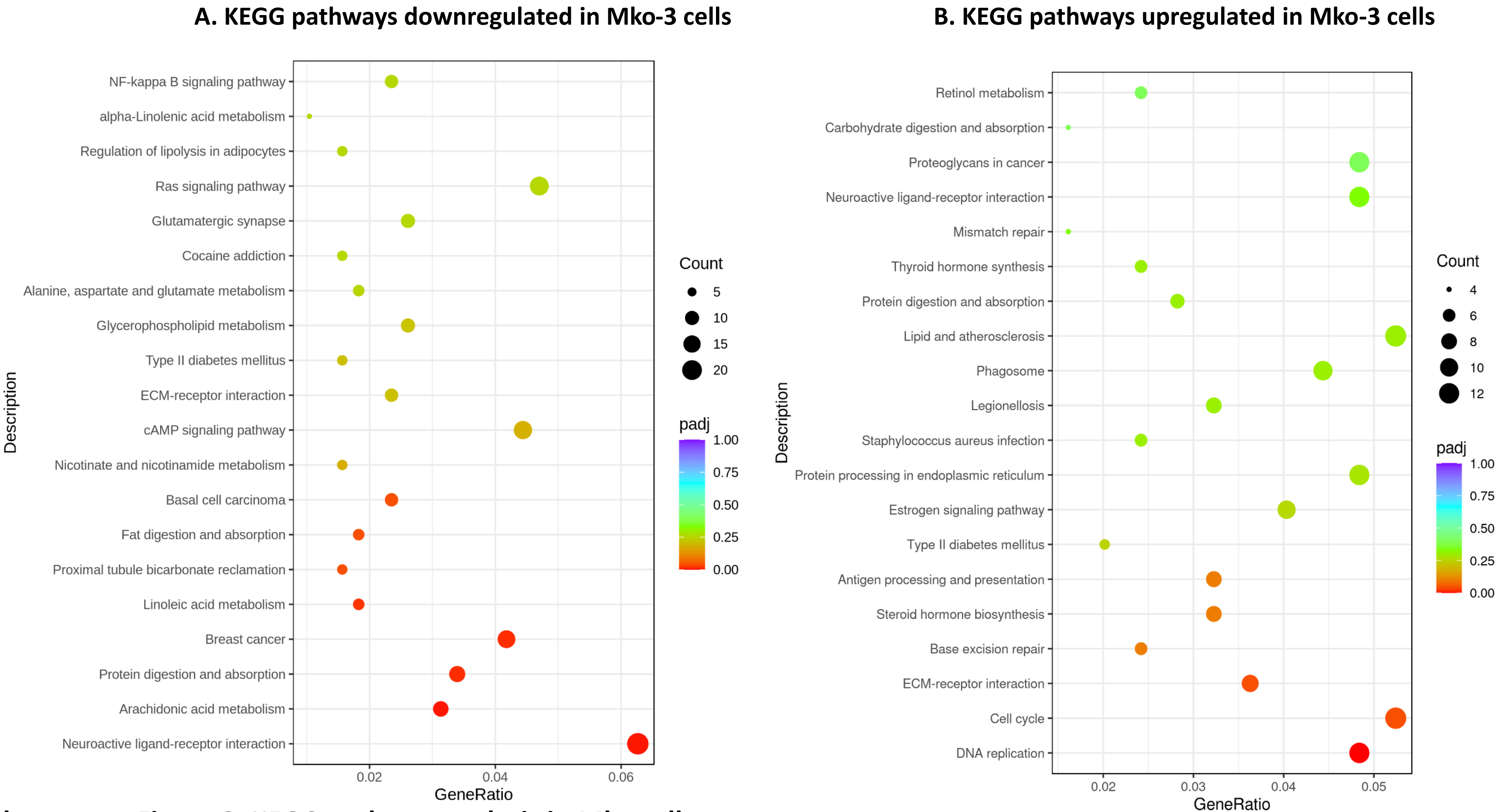

Supplementary Figure 8: KEGG pathway analysis in Mko cells

(A-B) KEGG enrichment analysis was performed to identify significantly upregulated and downregulated pathways in Mko cells.

Supplementary Figure 9

A. Gene Ontology Downregulated pathways in Mko-3 cells

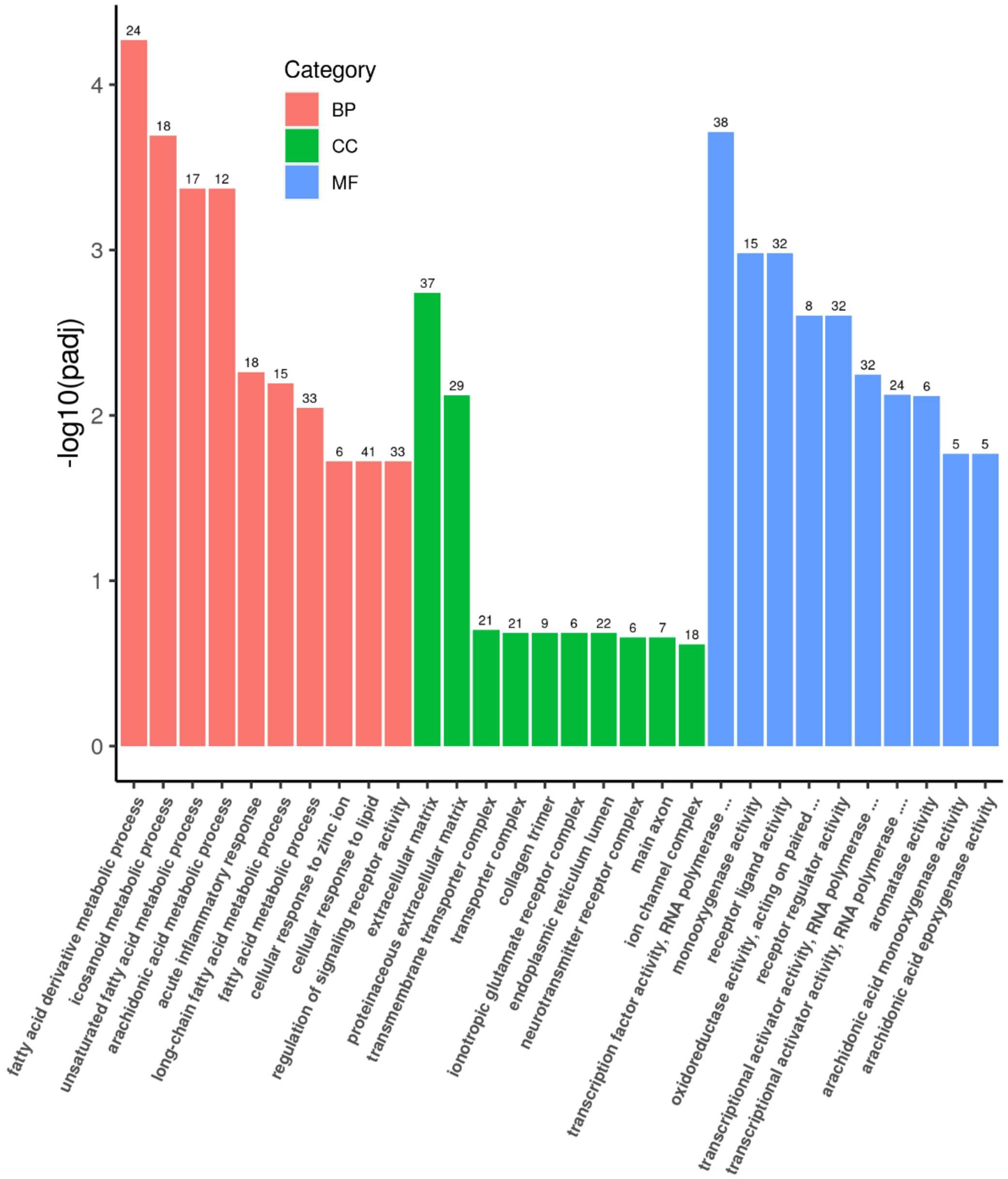

B. Gene Ontology Upregulated pathways in Mko-3 cells

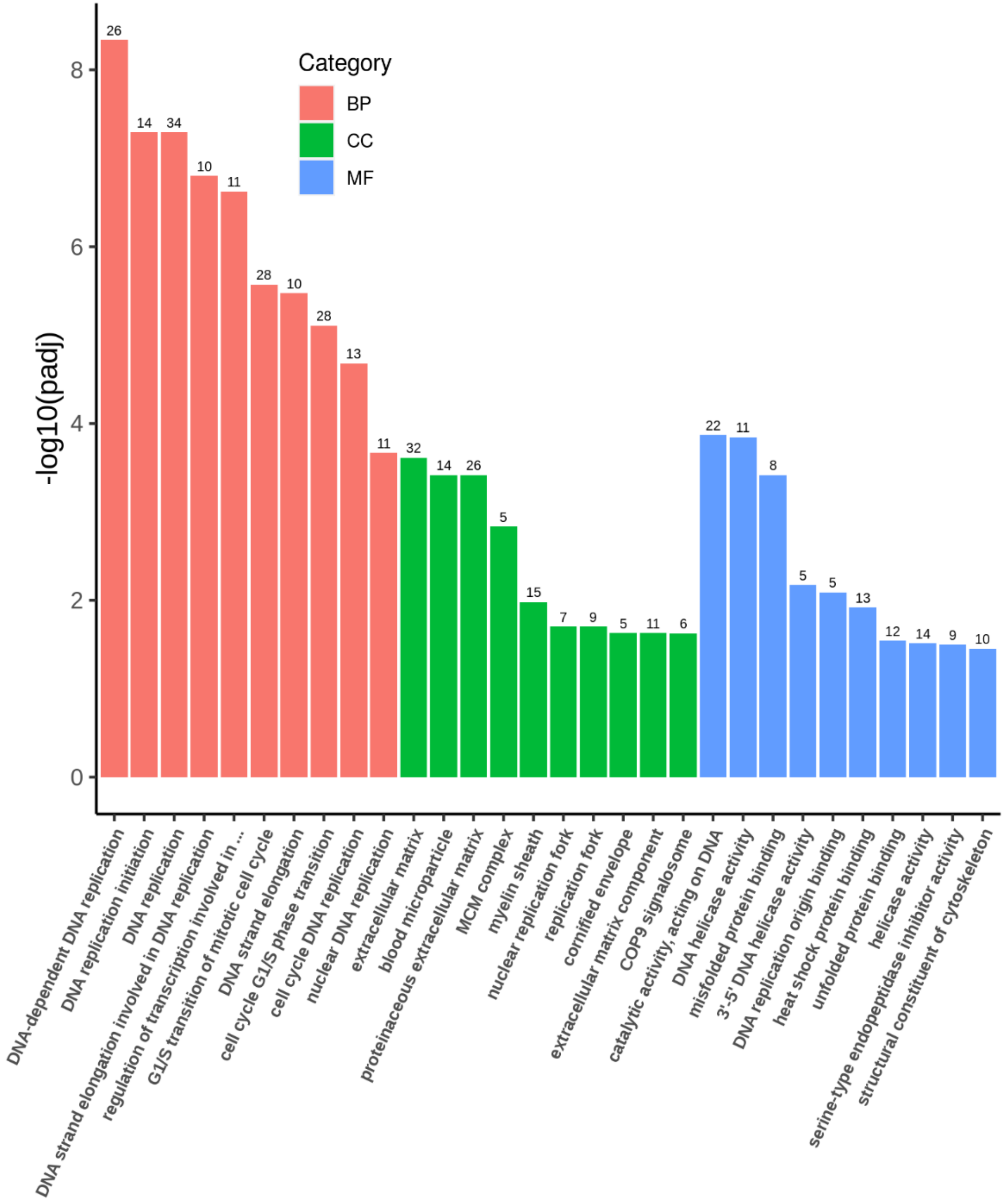

Supplementary Figure 9: Gene ontology in Mko cells
(**A-B**) Differentially expressed biological processes (BP), cellular component (CC), and molecular functions (MF) are identified in the Mko cells.
