# Supplementary material for: Human hepatoma Huh-7 cell culture models deficient in apolipoprotein B secretion
Source: J Lipid Res. 2025 Jul 23;66(8):100867. doi: 10.1016/j.jlr.2025.100867 (PMC12396022; doi:10.1016/j.jlr.2025.100867)
Supplement: Supplementray Tables [file mmc2.docx]

**Supplementary Table 1: Twenty most differentially expressed genes in Ako cells based on transcriptomic analysis**

| **Symbol** | **Gene name** | **Biological function** |
| --- | --- | --- |
| **GC** | GC, vitamin D binding protein | Multifunctional protein belonging to albumin gene family. Responsible for binding Vitamin D and its plasma metabolite and their transport to different organs (PMID: [31998239](https://pubmed.ncbi.nlm.nih.gov/31998239))(PMID: 31748273). |
| **TMSB4X** | Thymosin beta 4 X-linked | Actin sequestering protein which regulates actin polymerization. Also involved in cell proliferation, differentiation and migration (PMID: 38027718). |
| **MAGEA3** | MAGE family member A3 | It is highly expressed in different tumor types such as colon, melanoma, brain, lung, prostate and breast (PMID: [26342994](https://pubmed.ncbi.nlm.nih.gov/26342994)). |
| **MAGEC2** | MAGE family member C2 | Expression is restricted to testis but expressed in different tumor types (PMID: [24687377](https://pubmed.ncbi.nlm.nih.gov/24687377))(PMID: 30309319). |
| **PFKFB3** | 6-phosphofructo-2-kinase/fructose-2,6-biphosphatase 3 | Bifunctional protein is responsible for synthesis and degradation of fructose 2,6 bisphosphate, a regulatory step in glycolysis. (PMID: 29410405) |
| **TSPAN8** | Tetraspanin 8 | It is a cell surface glycoprotein and makes complex with integrins. It also mediates signal transduction pathways regulating cell development, activation, growth and motility. Highly expressed in pancreatic and colorectal cancer cells where it promotes cancer cell proliferation, migration and EMT transition (PMID: 36941633). Also identified to promote breast cancer progression (PMID: 35418219). |
| **CYP2B6** | Cytochrome P450 family 2 subfamily B member 6 | Expression is restricted to liver. Monooxygenase which catalyzes many reactions involved in drug metabolism and synthesis of cholesterol, steroids and other lipids (PMID: [27709010](https://pubmed.ncbi.nlm.nih.gov/27709010))(PMID: 36520866). |
| **MAGEA6** | MAGE family member A6 | Expression is restricted to testis, but it is also expressed in different tumor types (PMID: 35022469). |
| **ITIH2** | Inter-alpha-trypsin inhibitor heavy chain 2 | Plasma serine protease inhibitors play a role in stabilization of extracellular matrix and prevention of tumor metastasis. (PMID: [32639183](https://pubmed.ncbi.nlm.nih.gov/32639183))(PMID: 18226209). |
| **SLC43A3** | Solute carrier family 43 member 3 | Predicted to being involved in transmembrane transport. Regulates fatty acid flux in adipocytes (PMID: 32217606). |
| **COL2A1** | Collagen type II alpha 1 chain | Fibrillar collagen present in cartilage and vitreous humor of eye (PMID: [31824186](https://pubmed.ncbi.nlm.nih.gov/31824186)). |
| **SLC7A11** | Solute carrier family 7 member 11 | Member of heterodimeric sodium independent cysteine/glutamate antiporter known as system Xc which regulates ferroptosis. (PMID: 37339981)(PMID: 37537342). |
| **CTH** | Cystathionine gamma-lyase | An enzyme that converts methionine derived cystathione into cysteine thereby regulating glutathione production in liver. (PMID: 20191298) (PMID: 32305523). |
| **GDF15** | Growth differentiation factor 15 | Member of the TGF-β superfamily having a role in the recruitment and activation of SMAD transcription factors.  Expressed in different cell types and exhibits increased expression in response to cellular stress like hypoxia, mitochondrial dysfunction (PMID: 34381196). |
| **SH3BP2** | SH3 domain binding protein 2 | This protein binds to SH3 domain of different proteins and act as cytoplasmic adaptor protein to positively regulate the expression of various immune cells. Gene mutations have been associated with cherubism (PMID: 22640988). |
| **KIF21B** | Kinesin family member 21B | Belongs to kinesin protein superfamily. These are ATP dependent microtubule-based motor proteins with important roles in intracellular transport or membranous organelles (PMID: 37418324)(PMID: 30479371). |
| **JUN** | Jun proto-oncogene, AP-1 transcription factor subunit | It is a protooncogene and a member of dimeric activator protein 1 complex. It promotes tumorigenesis by regulating cell proliferation, migration senescence and metastasis (PMID: [22180088](https://pubmed.ncbi.nlm.nih.gov/22180088))(PMID: 23027969). |
| **AHR** | Aryl Hydrocarbon receptor | Ligand activated transcription factor with major role in mediating chemical toxicity. Studies suggest its involvement in microbial defense, inflammation and immunity and also in energy metabolism (PMID: 32535108)(PMID: [19538249](https://pubmed.ncbi.nlm.nih.gov/19538249)). |
| **SEMA6B** | Semaphorin 6B | Belongs to Semaphorin group of protein which have a conserved semaphoring domain and play major role in axon guidance. SEMA6B expression is reported to be downregulated by PPARα and its agonists. (PMID: 15177567). Other members of this protein are associated with liver fibrosis during chronic viral hepatitis C (PMID: [30592759](https://pubmed.ncbi.nlm.nih.gov/30592759)). |
| **CHAC1** | ChaC glutathione specific gamma glutamylcyclotransferase 1 | This protein promotes neural differentiation by regulating the notch signaling pathway (PMID: 27986595). it is also an important role in regulating glutathione levels and oxidative stress in the cell thereby regulating ferroptosis (PMID: 36675091). |

**Supplementary Table 2: Most significantly changed KEGG pathways and associated genes.**

| Pathway | Expression | Genes Involved |
| --- | --- | --- |
| MAPK signaling pathway | Downregulated | *JUN/FLNC/EPHA2/NFKB2/DDIT3/GADD45A/FGF19/RELB/FGFR1/*  *DUSP5/GADD45B/DUSP8/AREG/MYC/NR4A1/DUSP1/RAC2/EFNA3/*  *CACNB1/PLA2G4C/FGF17/JMJD7-PLA2G4B/FGF21/FGF2/CACNA1G/*  *EREG/CACNA2D2/PDGFB/MAP3K14-AS1/NFATC1* |
| Breast cancer | Downregulated | *JUN/NFKB2/GADD45A/FGF19/FGFR1/GADD45B/MYC/PIK3CD/WNT11/PRSS23/FGF17/FGF21/FGF2/DLL4/FZD9/DLL3/JAG2* |
| PI3K-Akt signaling pathway | Downregulated | *COL2A1/EPHA2/FGF19/FGFR1/CREB5/AREG/MYC/NR4A1/CSF3R/*  *PIK3CD/EFNA3/LPAR1/FGF17/FGF21/FGF2/CHAD/CDC37P2/LAMA4/*  *COL1A2/CDC37P1/AC234582.1/EREG/ITGA2B/GNG11/PDGFB/CSF3* |
| Melanoma | Downregulated | *GADD45A/FGF19/FGFR1/GADD45B/PIK3CD/FGF17/FGF21/FGF2/PDFB* |
| Amoebiasis | Downregulated | *CXCL8/C8B/PIK3CD/CXCL3/ARG2/IL12A/NOS2/LAMA4/COL1A2/IL12A-AS1* |
| Complement and coagulation cascades | Upregulated | *PROC/C1S/CPB2/A2M/SERPING1/C4BPA/PLG/MBL2/CFHR5/C7/CFHR2/C6/F8* |
| DNA replication | Upregulated | *LIG1/POLD3/FEN1/MCM5/MCM2/PCNA/MCM7/MCM4/POLE* |
| Chemical carcinogenesis - DNA adducts | Upregulated | *UGT2A3/GSTA1/AKR1C2/UGT2B4/SULT1A2/GSTO2/GSTA2/*  *CYP2C9/LINC02006/UGT2B11/UGT2B15* |
| Drug metabolism - cytochrome P450 | Upregulated | *CYP2B6/UGT2A3/GSTA1/UGT2B4/GSTO2/GSTA2/*  *CYP2C9/LINC02006/UGT2B11/ADH1C/UGT2B15* |
| Metabolism of xenobiotics by cytochrome P450 | Upregulated | *CYP2B6/UGT2A3/GSTA1/UGT2B4/GSTO2/GSTA2/*  *CYP2C9/LINC02006/UGT2B11/ADH1C/UGT2B15* |
| Chemical carcinogenesis - receptor activation | Upregulated | *CYP2B6/UGT2A3/GSTA1/UGT2B4/CDC25A/CDC6/GSTO2/GSTA2/E2F1/HSP90AA1/HSP90AA2P/LINC02006/UGT2B11/PAQR8/CHRNB2/UGT2B15* |
| Drug metabolism - other enzymes | Upregulated | *UGT2A3/GSTA1/UGT2B4/TK1/GSTO2/GSTA2/*  *XDH/RRM2/LINC02006/UGT2B11/UGT2B15* |
| Staphylococcus aureus infection | Upregulated | *C1S/PLG/DEFB1/MBL2/KRT17P8/DEFA9P/FCGR2B/DEFA8P* |
| Base excision repair | Upregulated | *UNG/LIG1/POLD3/FEN1/PCNA/POLE* |
| Steroid hormone biosynthesis | Upregulated | *UGT2A3/AKR1D1/AKR1C2/UGT2B4/HSD3B1/LINC02006/UGT2B11/UGT2B15* |
| Retinol metabolism | Upregulated | *CYP2B6/UGT2A3/UGT2B4/CYP2C9/LINC02006/UGT2B11/ADH1C/UGT2B15* |

**Supplementary Table 3: Most significantly changed proteins from proteomic analysis.**

| **Symbol** | **Gene name** | **Biological function** |
| --- | --- | --- |
| **GC** | GC, vitamin D binding protein | Multifunctional protein belonging to albumin gene family. Responsible for binding Vitamin D and its plasma metabolite and their transport to different organs (PMID: [31998239](https://pubmed.ncbi.nlm.nih.gov/31998239))(PMID: 31748273). |
| **BPHL** | Biphenyl hydrolase like | Hydrolytic enzyme belonging to serine protease family which facilitates the hydrolytic activation of antiviral prodrugs (PMID: 15832508). |
| **TNS1** | Tensin 1 | Tensin 1 is a focal adhesion molecule with multiple domains. It carries out actin polymerization and regulates several signal pathways due to presence of Src homology domain (PMID: 37011205). |
| **SETDB2** | SET domain bifurcated histone lysine methyltransferase 2 | Belongs to the protein family that contains methyl-CpG-binding domain (MBD) and s SET domain. It acts as a histone methyl transferase and mainly responsible for regulating chromosome segregation. |
| **ARHGAP44** | Rho GTPase activating protein 44 | Belongs to Rho GTPase activating family. Downregulation of its expression reduces tumor progression by targeting the p53/C-myc/Cyclin D1 pathway (PMID: [38031136](https://pubmed.ncbi.nlm.nih.gov/38031136)). |
| **MBD6** | methyl-CpG binding domain protein 6 | Member of methyl-CpG binding domain protein family which enables chromatin binding activity (PMID 24634419) (PMID: 25927341). Differential expression of this gene has been associated with uterine leiomyoma and BAP1 dependent tumor growth (PMID:31313936) (PMID: 36180891). |
| **BTBD11** | BTB (POZ) domain containing 11 | Primarily expressed in brain. Recent studies have explored its function in the hippocampal inhibitory neurons where its expression is correlated with anxiety related behavior (PMID: 37261953). |
| **OR4D10** | Olfactory Receptor Family 4 Subfamily D Member 10 | Member of the G-protein coupled receptor protein superfamily. |
| **GPR 158** | G-protein coupled receptor 158 | Orphan receptor of the GPCR protein family. It has been studied as a regulator of cell cycle progression and cell proliferation under glucocorticoid stimulated conditions (PMID: 23451275). |
| **SERPING1** | Plasma protease C1 inhibitor | The gene encodes protein C1 inhibitor which is a highly glycosylated plasma protein that regulates the complement cascade by inhibiting the activated C1r and C1s. Primarily synthesized in liver and its deficiency is associated with hereditary angioneurotic oedema (HANE) (PMID: [35958943](https://pubmed.ncbi.nlm.nih.gov/35958943)). |
| **APOB** | Apolipoprotein B | Structural component of the low density lipoproteins (LDL), very low density lipoproteins (VLDL) and chylomicrons. Major risk factor for cardiovascular and metabolic syndromes like diabetes and insulin resistance (PMID: [34677405](https://pubmed.ncbi.nlm.nih.gov/34677405)) (PMID: 34729547). |
| **RPAP3** | RNA polymerase II associated protein 3 | RNA polymerase II associated protein primarily involved in transcriptional regulation. Involved in the recruitment and positive regulation of HSP90 (PMID: 30033218). |
| **ADARB2** | Adenosine deaminase RNA specific B2 | RNA editing enzyme exclusively expressed in brain. Promotes tumor growth in glioblastomas and gain of function mutants have been reported in neuronal cells (PMID: 35922651)(PMID: 31552420). |
| **CES4A** | Carboxylesterase 4A | Belongs to carboxylesterase protein family with a role in the esterification and detoxification of xenobiotics. Also involved in fatty acyl and cholesterol ester metabolism (PMID: 28677105). |
| **EFCAB14** | EF-hand calcium binding domain 14 | Predicted to have a role in calcium ion binding (PMID: 32094379). |
| **MYO1C** | Myosin 1C | Myosin motor protein with a role in transcription initiation. It is responsible for actin polymerization and links it to cell membrane to regulate different cellular processes (PMID: 22908250) (PMID: 30872458). |
| **EPPK1** | Epiplakin 1 | Belongs to plakin family of proteins which are involved in cytoskeleton organization (PMID: 15671067). |
| **YLPM1** | YLP motif containing 1 | These proteins are predicted to regulate telomere maintenance and enable RNA binding activity. |
| **TRIM67** | Tripartite motif containing 67 | The TRIM family of proteins are involved in different cellular processes such as DNA repair, cell cycle regulation and apoptosis. Differential expression of TRIM67 is associated with non-alcoholic fatty liver disease (PMID: 35806477). Also plays tumor suppressive role by activating p53 signaling in colorectal cancer (PMID: 31239268). |
| **DHX15** | DEAH-box helicase 15 | It is an ATP dependent RNA helicase. Also act as an oncogene in Burkitts lymphoma by upregulation of NFκB pathway. (PMID: 35193582). |

**Supplementary Table 4: List of primers used for RT-qPCR**

| hApoB qPCR Forward Primer | CGCTAAAGGAGCAGTTGACC |
| --- | --- |
| hApoB qPCR Reverse Primer | TCACTGAAGACCGTGTGCTC |
| hMTP qPCR Forward Primer | TGTGGCCTTACTATGGAGGAA |
| hMTP qPCR Reverse Primer | AAG GAG CGT AGG TCT TTG CAG |
| hVDBP qPCR Forward Primer | GCCCAAAAAGTGCCTACTGC |
| hVDBP qPCR Reverse Primer | TACATCTGGAAGCTCGGGGA |
| SERPING1 qPCR ForwardPrimer | GCAACAACAGTGACGCCAAC |
| SERPING1 qPCR Reverse Primer | TGTTGTCTTCCACTTGGCACTC |
| C1S qPCR Forward Primer | ATCCAATGCCCTGCCCTAAG |
| C1S qPCR Forward Primer | ATGCCACAGTCCACAGGTTG |
| ATF4 qPCR Forward Primer | GGACTTGATGTCCCCCTTCG |
| ATF4 qPCR Reverse Primer | CGGAGAAGGCATCCTCCTTG |
| ATF6 qPCR Forward Primer | ACCTCCTTGTCAGCCCCTAA |
| ATF6 qPCR Reverse Primer | ACTCCCTGAGTTCCTGCTGA |
| BiP qPCR Forward Primer | CTTGCCGTTCAAGGTGGTTG |
| BiP qPCR Reverse Primer | TCTTTGGTTGCTTGGCGTTG |
| XBP1-u qPCR Forward Primer | AGGAGTTAAGACAGCGCTTGGGGATGGAT |
| XBP1-u qPCR Reverse Primer | CTGAATCTGAAGAGTCAATACCGCCAGAAT |
| XBP1-s qPCR Forward Primer | CCTGGTTGCTGAAGAGGAGG |
| XBP1-s qPCR Reverse Primer | CCATGGGGAGATGTTCTGGAG |
| 18S qPCR Forward Primer | GATCCGAGGGCCTCACTAAAC |
| 18S qPCR Reverse Primer | AGTCCCTGCCCTTTGTACACA |
| *APOB* Forward Primer (FP1) | TCTGCTTTTTCTTTCACGATCCC |
| *APOB* Reverse Primer (RP1) | GCAGGCTGTCCTCAAAGGTG |
|  |  |
